# Microfluidic Biochip‐Based Multiplexed Profiling of Small Extracellular Vesicles Proteins Integrated with Machine Learning for Early Disease Diagnosis

**DOI:** 10.1002/advs.202506167

**Published:** 2025-07-07

**Authors:** Xue Zhang, Yibin Jia, Zhikai Li, Yunhong Zhang, Chao Wang, Yanbo Liang, Jiaoyan Qiu, Mingyuan Sun, Xiaoshuang Chen, Miao Huang, Yu Zhang, Jianbo Wang, Hong Liu, Chuanbin Mao, Lin Han

**Affiliations:** ^1^ Institute of Marine Science and Technology Shandong University Binhai Road Qingdao Shandong Province 266237 China; ^2^ Department of Radiation Oncology Qilu Hospital Cheeloo College of Medicine Shandong University Wenhua Xi Road Jinan Shandong Province 250012 China; ^3^ Department of Biomedical Engineering The Chinese University of Hong Kong Sha Tin Hong Kong SAR China; ^4^ State Key Laboratory of Microbial Technology Shandong University Binhai Road Qingdao Shandong Province 266237 China

**Keywords:** early diagnosis, machine Learning, microfluidic platform, small extracellular vesicles proteins

## Abstract

Accurate early diagnosis is essential for preventing diseases and improving cure and survival rates. There are no reliable early‐diagnosis biomarkers for most major diseases. Here, esophageal squamous cell carcinoma (ESCC) is used as a disease model to develop a platform for detecting a panel of proteomic biomarkers for accurate early diagnosis by integrating a barcode immunoassay biochip with machine learning. The biochip captures small extracellular vesicles (EVs) from serum, lyses them in situ, and quantifies multiple proteins, including membrane and internal proteins of EVs. It is utilized to test 273 clinical samples across multiple centers. The validation sets are then analyzed using machine learning, resulting in a precise diagnostic model for ESCC. This model, based on nine diagnostic protein biomarkers identified through mass spectrometry analysis of differentially expressed proteins, achieves an accuracy of 91.0% in external validation, with a 90.8% accuracy in detecting early‐stage ESCC. These results significantly surpass the accuracy (only 14.4%) of the currently used biomarker for squamous cell carcinoma. Thus, integrating extracellular vesicles protein analysis with machine learning presents can identify ESCC patients. The developed extracellular vesicles analysis platform offers a promising tool for the clinical application of multi‐biomarker detection methods, advancing the early diagnosis of ESCC.

## Graphical abstract

This study develops a diagnostic platform for early detection of esophageal squamous cell carcinoma (ESCC) by integrating a barcode immunoassay biochip with machine learning. The biochip captures and analyzes small extracellular vesicle proteins from serum samples, identifying nine biomarkers linked to ESCC. Validation of 273 clinical samples achieves 91% accuracy, significantly outperforming existing diagnostic methods.

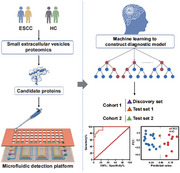

## 1 Introduction

Malignant tumors are one of the biggest killers of humans. For instance, esophageal cancer (EC) is ranked as the seventh most common malignant tumor globally, with squamous cell carcinomas accounting for 90% of cases and exhibiting a high mortality rate that significantly impacts patients' life quality.^[^ ^1^, ^2^ ^]^ While some traditional screening methods, for instance, endoscopic screening, reduce the incidence of EC, these methods are invasive, costly, and require a skilled operator.^[^ ^3^, ^4^, ^5^ ^]^ Additionally, tissue biopsies are limited by the challenges of obtaining samples and are subject to sampling bias due to the temporal and spatial heterogeneity of tumors.^[^ ^6^ ^]^ Consequently, less invasive methods are desired to screen a larger portion of the population.^[^ ^7^ ^]^

Liquid biopsies are minimally invasive, safe, and can overcome the difficulties associated with intra‐tumor heterogeneity.^[^ ^8^, ^9^ ^]^ Cell‐free DNA (cfDNA) and free proteins are the most commonly used markers for liquid biopsies; however, circulating cfDNA and free proteins have less than 20% sensitivity for early‐stage EC.^[^ ^10^, ^11^, ^12^ ^]^ Small extracellular vesicles, a subset of extracellular vesicles (EVs) smaller than 200 nm, are ubiquitously found in the interstitial space of tissues and body fluids, carrying molecular fingerprints indicative of their cellular origin.^[^ ^13^, ^14^ ^]^ Compared to other liquid biopsy markers, such as circulating tumor cells, small EVs are more abundant in blood, contain more diverse information, and are more stable due to their intact membrane structure.^[^ ^15^ ^]^ In various cancer types, including colorectal cancer (CRC),^[^ ^16^ ^]^ pancreatic ductal adenocarcinoma,^[^ ^17^, ^18^ ^]^ prostate cancer,^[^ ^19^ ^]^ and ovarian cancer,^[^ ^20^ ^]^ RNA and proteins carried by small EVs can serve as promising biomarkers, providing reliable information for tumor diagnosis and prognosis.

Small EVs membrane proteins are primarily involved in functions such as EVs formation, intercellular communication, and targeting specificity.^[^ ^21^ ^]^ In contrast, most proteins inside small EVs are derived from the physiological or pathological state of the parent cell. This allows the internal proteins of small EVs to reflect intracellular biological processes and signaling mechanisms, thereby offering valuable insights into the role of small EVs in intercellular information transfer.^[^ ^22^ ^]^ Therefore, a comprehensive analysis of both membrane and internal proteins of small EVs can yield more holistic and precise biological data, which is crucial for evaluating the overall physiological state of the organism. Mass spectrometry can screen both small EVs membranes and internal proteins and can identify thousands of proteins. However, it takes mass spectrometry days to conduct the detection, is costly, and needs a large amount of sample volume, which limits its applications in clinical large‐scale screening. The combination of mass spectrometry and other detection techniques is promising for clinical applications, since mass spectrometry provides a list of differentially expressed proteins, and another detection technique conducts the fast, economic, and sensitive detection of small volume of clinical samples. Biochips have gained recognition for their advantages in detection speed, economic cost, and low sample consumption.^[^ ^23^, ^24^ ^]^ Currently, small EVs capturing platforms based on biochips demonstrate high specificity and sensitivity.^[^ ^25^, ^26^, ^27^, ^28^, ^29^ ^]^ However, existing biochip platforms for small EVs marker detection primarily focus on small EVs surface proteins, lacking a rapid platform for the comprehensive detection of both the membrane and internal proteins of small EVs.

Here, using esophageal squamous cell carcinoma (ESCC) as a model, we developed an small EVs proteins detection platform integrating mass spectrometry and a barcode immunoassay biochip to investigate both membrane and internal proteins in patients and identify protein markers (differentially expressed in cancer patients and healthy control) with diagnostic potential through serum small EVs proteome. The mass spectrometry identified over 2000 proteins and provided potential protein markers for early diagnosis. The biochip captures small EVs from serum, lyses them in situ, and quantitatively analyzes multiple potential protein markers identified by mass spectrometry in clinical samples from two cohorts. Then, a reliable diagnostic model is developed for ESCC based on machine learning algorithms, which is validated in an independent clinical cohort. This platform enables high‐throughput and rapid analysis of multiple samples from multi‐centers, facilitating the translation of multi‐biomarker assays into clinical applications.

## 2 Results

### 2.1 Design of Small EVs Proteins Analysis for Early Diagnosis

**Figure 1:**
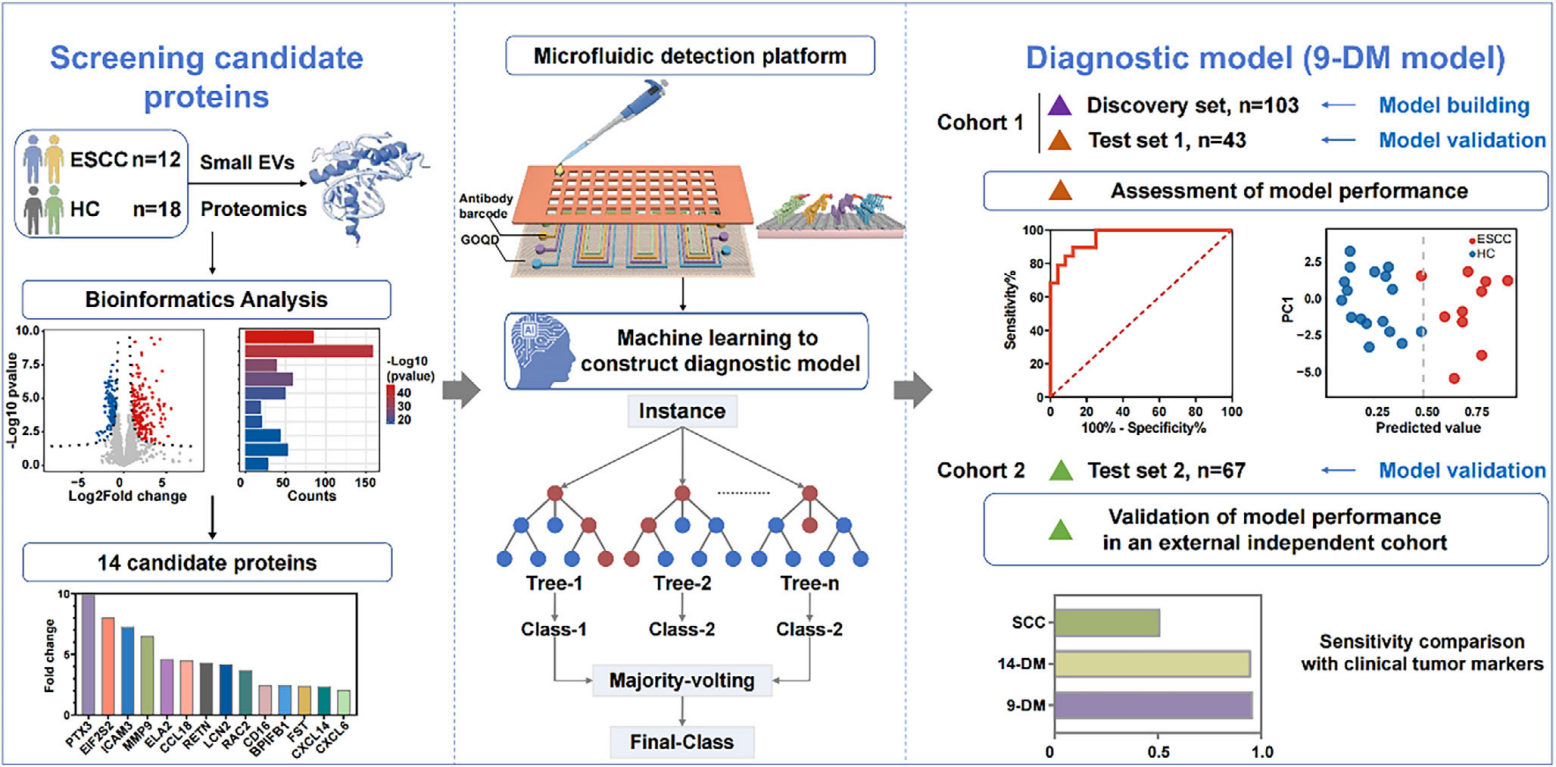
Schematic diagram of the small EVs protein panel for ESCC diagnosis. Abbreviations. DM: diagnostic markers; ESCC: esophageal squamous cell carcinoma; EVs: extracellular vesicles; HC: healthy controls; PC: principal component.

The general process of this study, including specific details on participant recruitment for each analysis, is presented in **Figure** **1**. In particular, we employed a liquid chromatography‐mass spectrometry (LC‐MS)‐based 4D data‐independent acquisition (4D‐DIA) method on 12 ESCC patients and 18 healthy controls (HC) to obtain proteomic data from their serum small EVs. Over 2000 small EVs proteins were recognized, and 14 potential markers were screened according to their differential expression and bioinformatics significance. The expression of these markers was then compared between 66 ESCC patients and 80 HC in Cohort 1. Machine learning techniques were utilized to explore the correlation between protein profiles and clinical phenotypes. A diagnostic model for ESCC was developed, and 9 of 14 potential markers were optimized to build the 9‐DM model, and its performance was evaluated in distinguishing ESCC patients from HC. Additionally, an external test set was employed to verify the robustness of the machine learning model, which has 67 samples, including 47 ESCC patients and 20 healthy persons.

### 2.2 Screening of Candidate Small EVs Protein Markers

**Figure 2:**
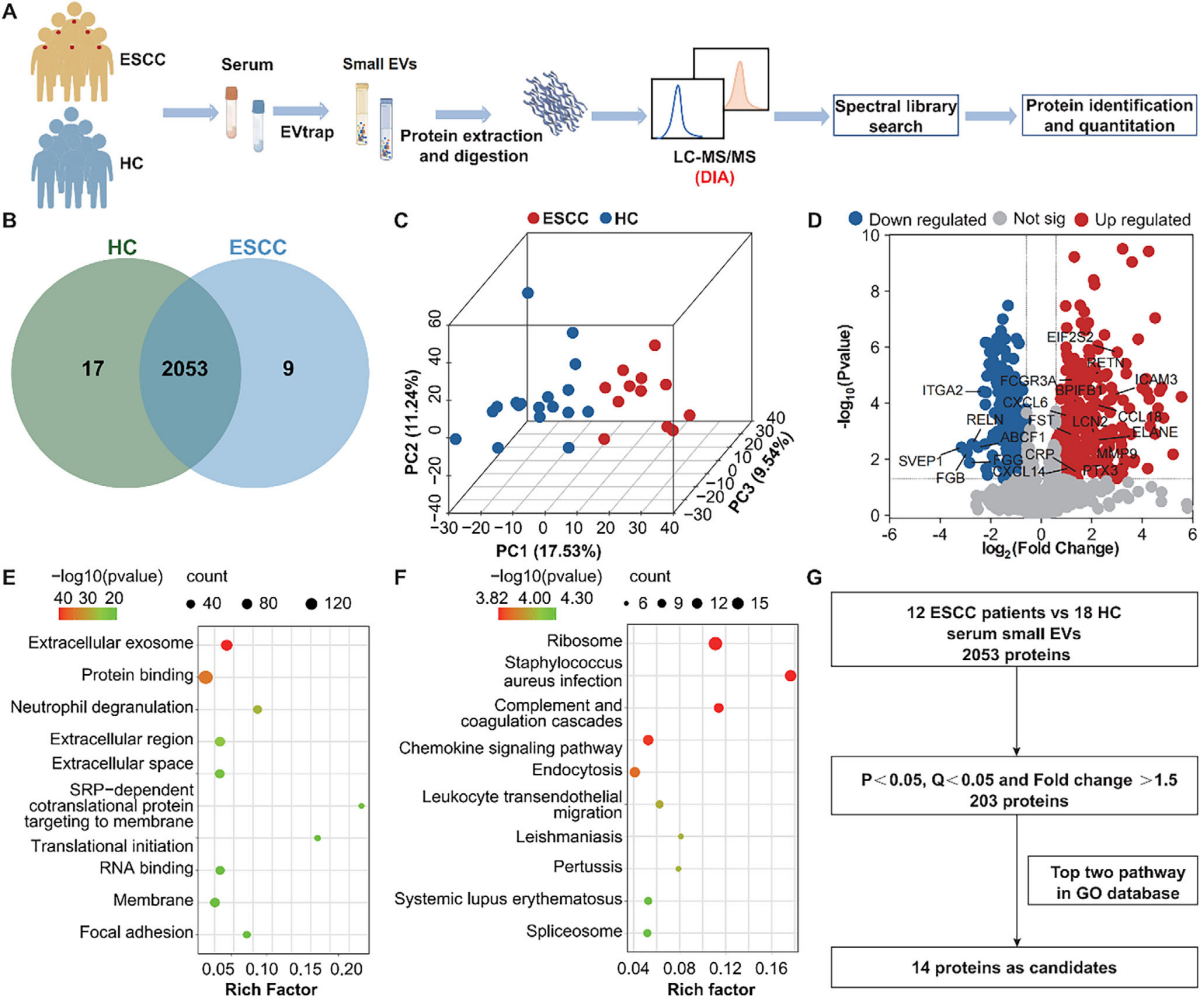
Proteomic landscape for ESCC and diagnosis relevance of functional protein modules. A) Flow chart of serum small EVs mass spectrometry. B) Venn diagram showing the overlap between the proteins identified in ESCC and HC serum small EVs. C) PCA plot displaying analyzable proteins in ESCC and HC serum small EVs. Each dot represents a sample, with blue dots for HC samples and red dots for ESCC samples. The % value indicates the explained variance. D) Volcano plot displaying differentially abundant proteins between ESCC and HC. Each dot represents a protein, with red dots for proteins significantly upregulated in ESCC and blue dots for proteins significantly downregulated in ESCC. The significance threshold is defined as upregulated in tumors (adjusted *P* < 0.05, log2FC > 0.58 (FC > 1.5)), downregulated in tumors (adjusted *P* < 0.05, log2FC < −0.58), or otherwise considered not significant. *P* values were calculated using the R package “limma” and adjusted using the Benjamini–Hochberg method. Two‐sided *P* values were calculated. E) Bubble plots showing the GO and F) KEGG pathway enrichment of ESCC and HC groups. The adjusted *P* < 0.05 is considered statistically significant. *P* values were calculated from KOBAS and adjusted using the Benjamini–Hochberg method. Two‐sided *P* values were calculated. Abbreviations. ESCC: esophageal squamous cell carcinoma; HC: healthy controls; PC: principal component; PCA: principal component analysis; Not sig: not significant.

To characterize the protein function of serum small EVs in ESCC, we performed 4D‐DIA mass spectrometry on small EVs isolated from the serum samples of 12 patients with ESCC and 18 HC (**Figure** **2**A). In total, 2062 proteins were identified in the serum small EVs of ESCC patients, while 2370 proteins were detected in HC, among which 2053 proteins are common to both groups (Figure 2B). Principal component analysis (PCA) effectively separated ESCC samples from HC, suggesting that the serum small EVs proteome of ESCC underwent significant remodeling (Figure 2C).

The differences between the common serum small EVs proteins of ESCC and HC were further analyzed using a fold change (FC) threshold of ≥1.5 and a significance level of *P* < 0.05. This analysis reveals that 203 proteins in the serum small EVs of ESCC are significantly upregulated compared to those in HC (Figure 2D). Additionally, Gene Ontology (GO) analysis of the differential proteins reveals that proteins in the serum extracellular vesicles of ESCC patients are significantly enriched in the pathways related to “Extracellular exosome,” “Protein binding,” and “Neutrophil degranulation” (Figure 2E). Furthermore, Kyoto Encyclopedia of Genes and Genomes (KEGG) pathway indicates significant upregulation in the “Ribosome”, “Chemokine signaling pathway” and “Endocytosis” pathways (Figure 2F). The above findings illustrate that multiple significant functional and metabolic pathways are activated in the serum small EVs of patients with ESCC. Based on the analysis of the serum small EVs proteomic differences between ESCC patients and HC, along with the top two GO pathways, 14 small EVs proteins are selected, which are significantly elevated in ESCC as candidate markers for subsequent diagnostic analysis (Figure 2G; Figures S1 and S2, Supporting Information).

### 2.3 Barcode Immunoassay Chip for Serum Small EVs Proteins Screening

**Figure 3:**
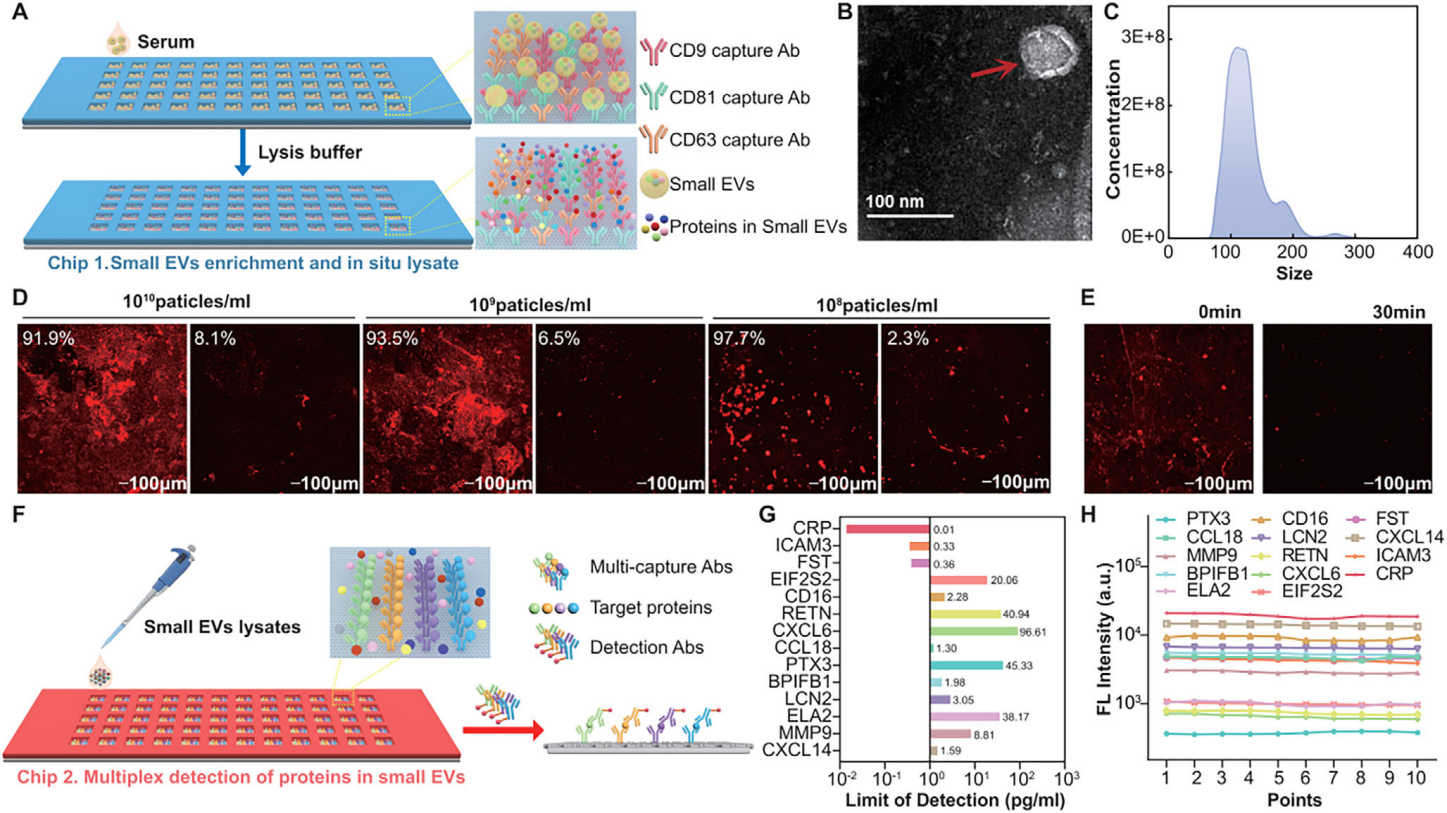
Small EVs analysis platform. A) Schematic of the chip for small EVs capture and in situ lysis. Capture antibodies for transmembrane proteins of small EVs are immobilized on a self‐assembled GOQD substrate to facilitate small EVs capture. Subsequently, in situ lysis of the small EVs is performed. This method enables the simultaneous detection of 60 samples, with each requiring 10 µL of serum. B) Representative transmission electron microscopy images of small EVs isolated with a lipid bilayer and cup‐shaped structure. Scale bar‐100 nm. C) Particle size distributions of small EVs measured by nanoparticle tracking analysis. D) Fluorescence scanning images demonstrate the chip's capture capacity at concentrations ranging from 10^8^ to 10^10^ particles mL^−1^. Captured small EVs are detected using CD63 antibodies conjugated with APC, and scanning is performed with a fluorescence scanner with a 635 nm laser source. Calculation formula: [Initial fluorescence intensity/(Initial fluorescence intensity + Recovered fluorescence intensity)] ×100%. E) Fluorescence scanning images depict the lysis of small EVs at a concentration of 10^9^ particles mL^−1^. F) Schematic of the barcode immunoassay chip. The chip is constructed using GOQD‐assembled glass slides and a 60‐well PDMS microchamber layer. Antibodies for target proteins are micro‐patterned in a specified order to specifically capture target proteins from small EVs lysates. After incubation with detection antibodies conjugated with APC, fluorescence scanning is performed. G) The bar chart illustrates the detection limit of the 14 proteins. LOD = 3 ×δ /S, where δ refers to the relative standard deviation of the blank value and S refers to the slope in the linear regression equation. H) The scatter plot displays the stability of fluorescence readings for 14 proteins at a concentration of 1 ng mL^−1^. Fluorescence reading spots were randomly taken on the barcode. Abbreviations. Ab: antibody; GOQD: Graphene oxide quantum dots; LOD: limit of detection.

Efficient capture and detection of small EVs are crucial for the application of small EVs biomarkers. A barcode microfluidic platform is developed, which integrates small EVs capture, in situ lysis, and EV proteins detection to comprehensively analyze proteins both on the surface and inside small EVs, thereby improving the sensitivity of marker detection. Graphene oxide quantum dots (GOQDs) have demonstrated their strong capability to bind proteins and nucleic acids.^[^ ^23^, ^30^, ^31^ ^]^ Consequently, a polydimethylsiloxane (PDMS) chip with 60 reaction units is fabricated, and it is aligned with a GOQDs self‐assembled glass substrate.^[^ ^31^ ^]^ CD63, CD81, and CD9 are commonly used tetraspanin of small EVs that represent overlapping but distinct populations of small EVs.^[^ ^22^, ^24^, ^25^, ^26^ ^]^ As a result, a mixture of antibody CD63, CD81, and CD9 is immobilized on the GOQDs substrate in each reaction unit in order to improve the capture efficiency of small EVs (**Figure** **3**A). Then, serum samples are loaded into each reaction unit, and small EVs are captured on the chip. Transmission electron microscopy (TEM) and nanoparticle tracking analysis (NTA) of the small EVs are shown in Figure 3B,C and Figure S3 (Supporting Information). The size of small EVs is primarily concentrated below 200 nm. The capture efficiency of CD63, CD81, CD9 mixed antibodies is compared to that of three individual capture antibodies, and the mixed antibodies captured the largest number of small EVs both for cell line supernatant and serum (Figure S4A,B, Supporting Information), prompting us to use mixed antibodies for small EVs capture.

To evaluate the small EVs capture efficiency, a series of different small EVs concentrations were introduced into the reaction units. The small EVs were labeled by APC fluorescence, and the fluorescence signals were detected, which increased progressively from 10^5^ to 10^10^ particles mL^−1^ (Figure S4C, Supporting Information). By analyzing the fluorescence values between the captured small EVs and small EVs in the residue liquid, the small EVs capture efficiency is calculated, which reaches 91.9% at 10^10^ particles mL^−1^, 93.5% at 10^9^ particles mL^−1^, and 97.7% at 10^8^ particles mL^−1^ (Figure 3D). Through three independent experimental replicates, the platform consistently demonstrated EVs capture efficiencies exceeded 90% at concentrations ranging from 10^8^ to 10^9^ particles mL^−1^ (Figure S5, Supporting Information). Considering the typical small EVs concentration of ≈10^9^ particles mL^−1^ in healthy humans, a concentration of 10^9^ particles mL^−1^ from a cell line is utilized to optimize the incubation time. The amount of captured small EVs reaches the peak at 60 min of incubation at room temperature, and does not significantly improve the fluorescence signal of small EVs with extended incubation time (Figure S4D, Supporting Information). As a result, the optimal incubation time for small EVs is 60 min. After capturing the small EVs, in situ lysis of small EVs is performed, and the amount of small EVs remaining on the chip is evaluated at different lysis times. Radioimmunoprecipitation assay (RIPA) buffer and protease inhibitors were added to the reaction units containing APC‐CD63 small EVs on the chip, and the decrease in fluorescence signal reflected the lysis process (Figure 3E). It is observed that the fluorescence intensity of small EVs gradually weakened over time, converging to a background level after 30 min of lysis (Figure S4E, Supporting Information). As a result, 30 min are used to lyse captured small EVs.

Subsequently, we constructed an antibody barcode chip by microprinting the capture antibodies for the target proteins onto the GOQD substrate. First, a PDMS chip with 14 parallel microchannels is fabricated by soft photolithography,^[^ ^27^ ^]^ and it is aligned with a GOQDs self‐assembled glass substrate,^[^ ^27^ ^]^ enabling the simultaneous printing of at least 14 capture antibodies barcode for capture of corresponding target small EVs proteins (Table S1, Supporting Information). After capture antibodies barcode is printed on the GOQDs substrate, the microchannels PDMS chip is removed, and a PDMS chip for sample loading is aligned with the barcode chip (Figure 3F). The sample loading chip has 60 reaction units, and each unit covers a whole barcode array, which enables the simultaneous detection of 60 samples and 14 proteins of each sample. The lysis products were transferred to the antibody barcode chip for small EVs proteins detection, and the fluorescence values derived from the antibody barcode were recorded (Figure S4F, Supporting Information). Furthermore, in control chips coated with BSA but without EVs‐capture antibodies, no specific signals of the target biomarkers were detected (Figure S6, Supporting Information), demonstrating that our chip system achieved specific EVs capture while effectively preventing nonspecific binding of non‐vesicular components. Furthermore, the GOQD‐functionalized chips exhibited stable performance, demonstrating < 5% RSD in fluorescence signals during 24‐week storage at 4 °C. Antibody‐conjugated GOQD chips showed comparable stability with < 10% signal attenuation during 10‐month storage at −20 °C (Figure S7, Supporting Information). First of all, 14 recombinant proteins at various concentrations are detected, and their quantitative curves are plotted as in Figure S8 (Supporting Information). They present excellent specificity with each other (Figure S9, Supporting Information). For the 14 small EVs proteins, the chip showed a detection limit of 0.01–96.61 pg mL^−1^ (Figure 3G). To assess the uniformity of the barcode chip readings, the protein concentration was standardized at 1 ng mL^−1^, and the 10 spots were randomly selected from the barcode fluorescent strip, which showed an error range of 2–7% of the mean reading value (Figure 3H).

### 2.4 Barcode Immunoassay Platform‐Based Clinical Sample Testing

**Figure 4:**
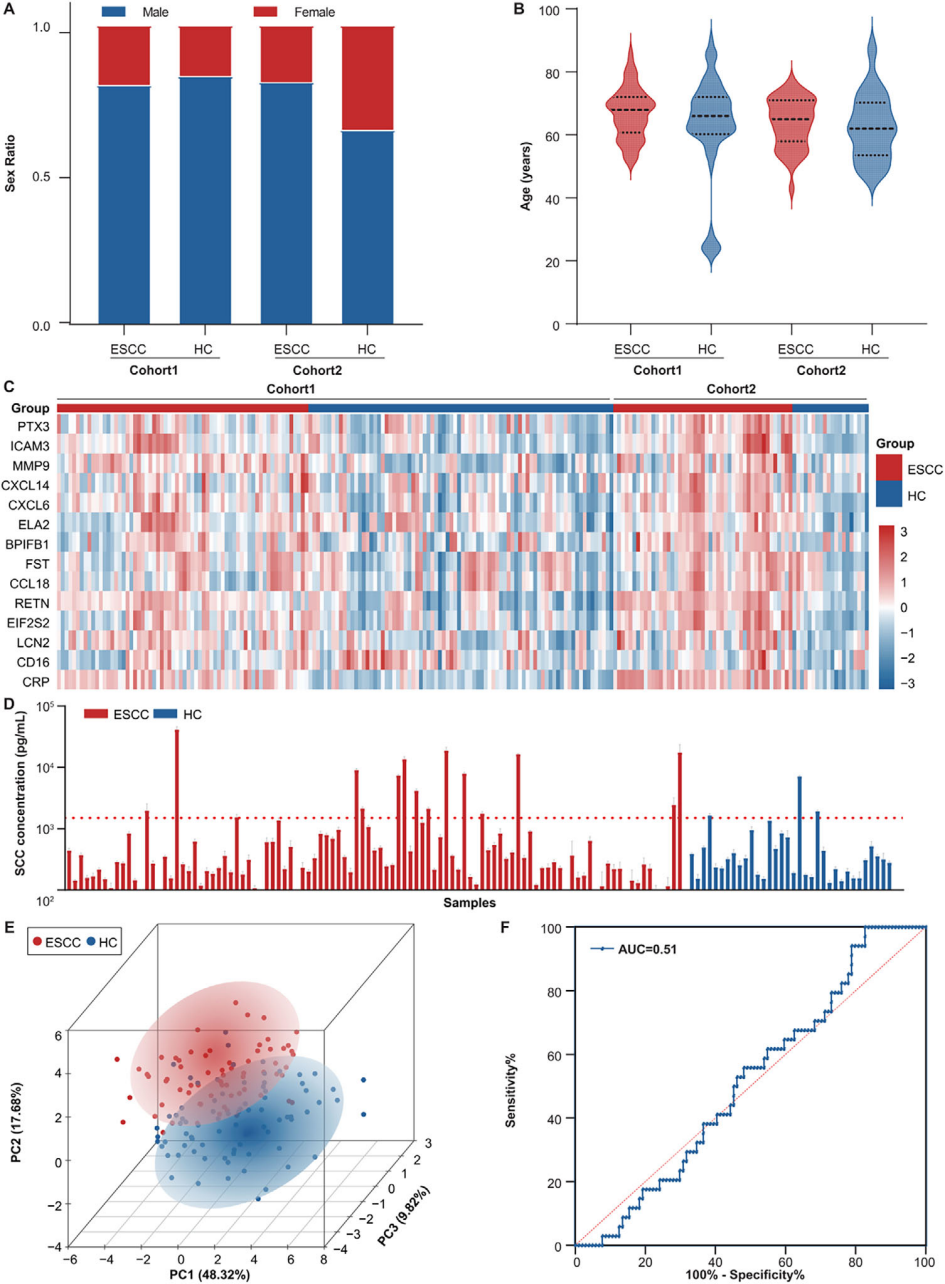
14 candidate diagnostic markers and SCC detection based on a microfluidic platform. A) The bar chart presents the gender ratio of ESCC and HC in two cohorts. B) The violin plot illustrates the age distribution of ESCC and HC in two cohorts. C) The heatmap displays the relative abundance of 14 proteins in Cohort 1 and Cohort 2. D) The bar chart presents the concentration of SCC in the serum of ESCC patients and HC. Each bar represents an individual sample, with red indicating ESCC and blue indicating HC. E) PCA plot displaying 14 proteins in ESCC and HC serum small EVs. Each dot represents a sample, with blue dots for HC samples and red dots for ESCC samples. The % value indicates the explained variance. F) ROC curve for SCC used to differentiate between ESCC and HC. Abbreviations. ESCC: esophageal squamous cell carcinoma; HC: healthy controls; PC: principal component; PCA: principal component analysis; AUC: area under the curve.

To validate the consistency between our microfluidic chip and mass spectrometry results, we performed parallel detection of the 14 candidate proteins in the same ESCC patient and healthy control cohorts using both platforms. Comparative analysis demonstrated strong concordance in differential expression patterns for the majority of proteins across platforms (Figures S10 and S11, Supporting Information). Based on the barcode immunoassay platform described above, we examine the 14 candidate proteins in the serum small EVs from Cohort 1 and Cohort 2. First, the two cohorts of ESCC patients and HC have a similar sex ratio (**Figure** **4**A; Figures S12A,C, Supporting Information), and the age distribution is predominantly over 50 years (Figure 4B; Figure S12B,D, Supporting Information). Thus, potential interference due to age and gender differences between groups could be excluded. Additionally, there are no significant differences in the basic clinicopathologic characteristics of ESCC patients between the two cohorts (Figure S12E, Table S2, Supporting Information), indicating that the two clinical cohorts are comparable.

The levels of these 14 proteins in the serum small EVs of ESCC patients in Cohorts 1 and 2 show significant differences compared to those of HC (Figure 4C). Specifically, the mean expression levels of PTX3, ICAM3, MMP9, CXCL6, BPIFB1, FST, RETN, EIF2S2, LCN2, and CRP are significantly higher in ESCC patients (Figures S13–S16, Supporting Information).

To further assess whether the markers screened offer advantages over existing clinical markers, the expression levels of SCC were detected, which is a commonly used squamous cell carcinoma marker. Using the microfluidic platform, the fluorescence intensity of standard proteins was measured at varying concentrations of SCC to create a standard protein curve (Figure S17A, Supporting Information). The expression levels of SCC in the serum were then measured in 104 ESCC patients and 34 healthy controls (Figure S17B, Supporting Information). And the concentration was calculated according to the measured fluorescence intensity and the standard protein quantitative curve. It is found that only 14.4% of ESCC patients have SCC expression levels exceeding 1.5 ng mL^−1^ (Figure 4D). Furthermore, compared to HC, the average expression level of SCC in ESCC patients did not show a significant difference (Figure S17C, Supporting Information).

Additionally, PCA based on the fluorescence levels of the 14 screened proteins successfully distinguishes a more pronounced subgroup of ESCC patients from HC (Figure 4E). The diagnostic efficacy of the markers is evaluated using ROC curves, with the area under the ROC curves (AUC) for individual protein markers ranging from 0.52 to 0.88 (Figure S18, Supporting Information), while the AUC for SCC was only 0.51 (Figure 4F). In conclusion, the 14 markers identified in this study demonstrate potential clinical diagnostic value for ESCC.

### 2.5 Construction and Validation of a Precise Diagnostic Model

**Figure 5:**
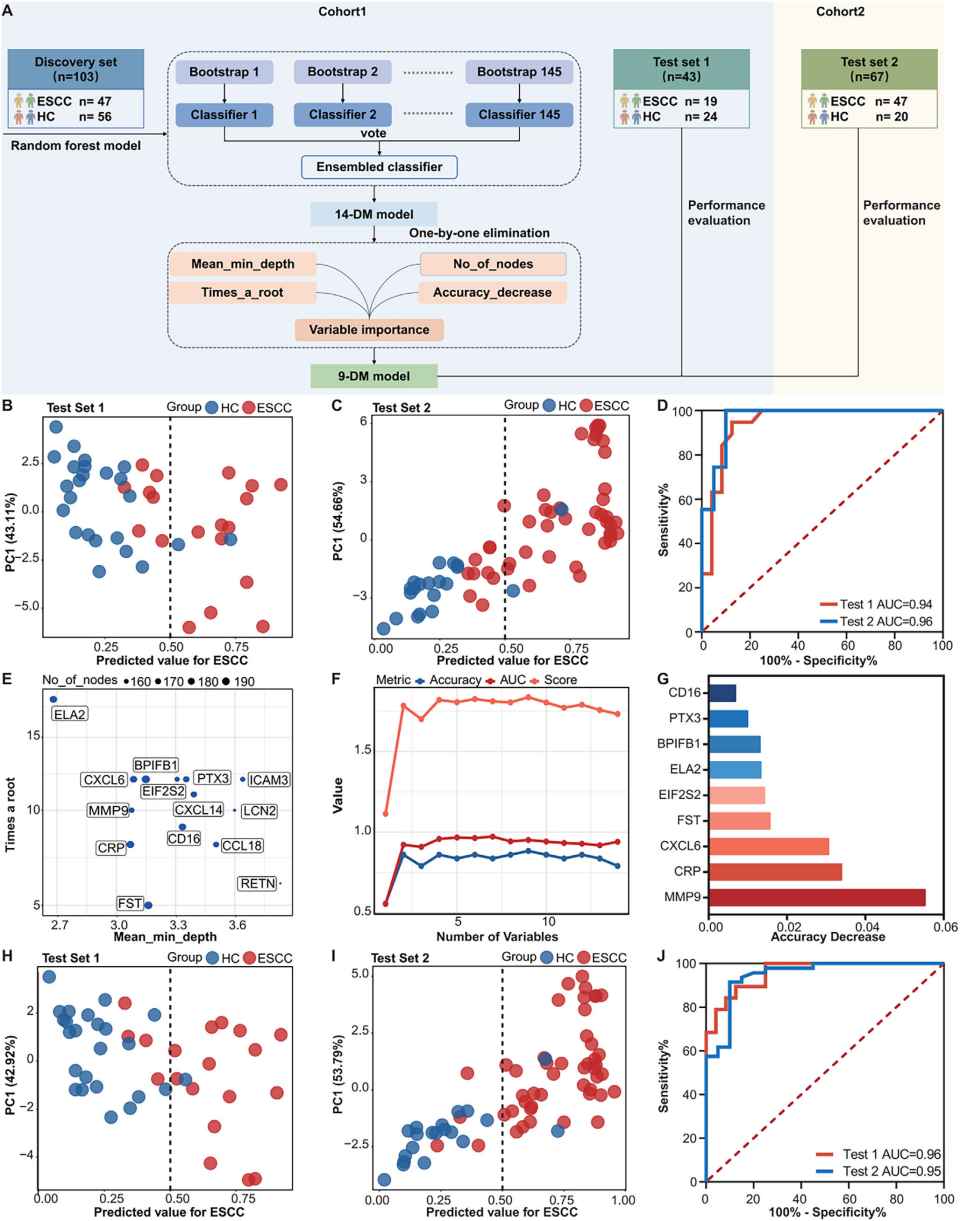
Machine learning‐derived prediction model based on serum small EVs proteins for ESCC diagnosis. A) Workflow design for constructing the diagnostic model. Feature selection and model training were performed using the random forest algorithm. The 14‐DM and 9‐DM models were validated in the internal test set (Test Set 1) and the external test set (Test Set 2). B) Predictive performance of the 14‐DM model in distinguishing ESCC (red) from HC (blue) in test set 1, and C) test set 2. The dashed line indicates a cutoff value of 0.50 for separately predicting HC (left) and ESCC (right). D) ROC curves for the 14‐DM model in diagnosing ESCC in test sets 1 and 2. E) Importance distribution plot of 14 biomarkers. Each point represents a protein biomarker, with the size of the point indicating the total number of nodes where the biomarker is used for splitting in the model. The *X*‐axis represents the average minimum depth of the variable, with smaller values indicating greater importance, while the *Y*‐axis represents the total number of trees in which the variable is used for splits. F) Line graph illustrating the model accuracy, AUC, and combined score of accuracy and AUC as different numbers of variables are included. G) Bar chart displaying the decline in accuracy of the 9‐DM model upon removal of each variable. H) The 9‐DM model predicts ESCC (red) versus HC (blue) in test sets 1 (G) and I) test 2. The dashed line indicates a cutoff value of 0.50 for separately predicting HC (left) and ESCC (right). J) ROC curves for the 9‐DM model in diagnosing ESCC in test set 1 and set 2. Abbreviations. AUC: area under the curve; DM: diagnostic markers; ESCC: esophageal squamous cell carcinoma; HC: healthy controls; PC: principal component.

In order to develop an innovative diagnostic method for ESCC using candidate proteins, machine learning is employed to create models that precisely predict clinical status (**Figure** **5**A). Initially, a model is constructed based on the 14 proteins using a random forest algorithm, and model parameters are optimized based on the combined scores of accuracy (ACC) and AUC (Figure S19A–C, Supporting Information). The optimal values were set at ntree = 145 and mtry = 1.

Cohort 1 was randomly split into a training set and a test set at a ratio of 7:3, while Cohort 2 served as an independent external test set, referred to as test set 2. To visualize the model performance, the graphs were generated to compare predicted values against actual disease status (ESCC or HC). With a cutoff value of 0.5 for classification, the 14‐diagnostic markers (DM) model accurately identified 63.2% of ESCC patients in test set 1 and 78.7% in test set 2 (Figure 5B,C). The model demonstrated 100% accuracy in the training set (Figure S19D, Supporting Information), with accuracy rates of 79.1% in test set 1 (Figure S19E, Supporting Information) and 82.1% in test set 2 (Figure S19F, Supporting Information). The AUC values were 0.94 and 0.96 for the respective test sets (Figure 5D).

To further optimize the model and reduce detection costs, the importance of variables in the 14‐DM model was ranked using the random forest algorithm. ELA2, CXCL6, MMP9, CRP, and BPIFB1 were ranked in the top five based on the average minimum depth and node counts (Figure 5E), and the other 9 proteins were applied to further analysis. As a result, the 9 protein variables yield the highest combined score based on ACC, AUC, and overall performance (Figure 5F). The selected variables are MMP9, CRP, CXCL6, FST, EIF2S2, ELA2, BPIFB1, PTX3, and CD16. Excluding the variable one by one results in varying degrees of accuracy decline, and it is observed that MMP9 presents the most significant decrease (Figure 5G), which means MMP9 plays an important role in the diagnostic model.

An improved random forest model is constructed using the nine variables, optimizing parameters with n tree set to 400 and mtry to 1 (Figure S20A–C, Supporting Information). This 9‐DM model accurately identifies 79.0% of ESCC patients in test set 1 and 91.5% in test set 2 (Figure 5H,I). Accuracy rates are 88.4% in test set 1 (Figure S20D, Supporting Information) and 91.0% in test set 2 (Figure S20E, Supporting Information), with AUC values of 0.96 and 0.95 for the respective test sets (Figure 5J). These results indicate that the 9‐DM model exhibits high sensitivity and reliability.

To further validate the generalizability of the selected biomarkers and the 9‐DM model, an independent validation cohort (Cohort 3) consisting of 30 ESCC patients and 30 healthy controls was recruited at Qilu Hospital between January and March 2025. External validation results demonstrated consistent expression patterns of most candidate biomarkers with those observed in Cohorts 1 and 2 (Figures S21 and S22, Supporting Information), while the 9‐DM model maintained robust diagnostic performance with 83.3% classification accuracy (AUC = 0.93) (Figure S23, Supporting Information), thereby confirming the clinical utility and reliability of our protein signature for ESCC detection.

To assess its effectiveness in diagnosing early‐stage ESCC, the 9‐DM model is applied to differentiate between stage I‐II ESCC and HC in both test sets. The model successfully identified 85.7% of early ESCC patients and 93.2% of HC, resulting in an overall accuracy of 90.8% (Figure S20F,G, Supporting Information) and an AUC of 0.96 (Figure S20H, Supporting Information). In clinical settings, early detection of ESCC is vital for prompt intervention and radical resection, which can reduce surgical difficulties and significantly improve patient survival rates.^[^ ^28^, ^29^ ^]^

### 2.6 The Specificity of Small EVs Protein Markers to Differentiate Cancers

**Figure 6:**
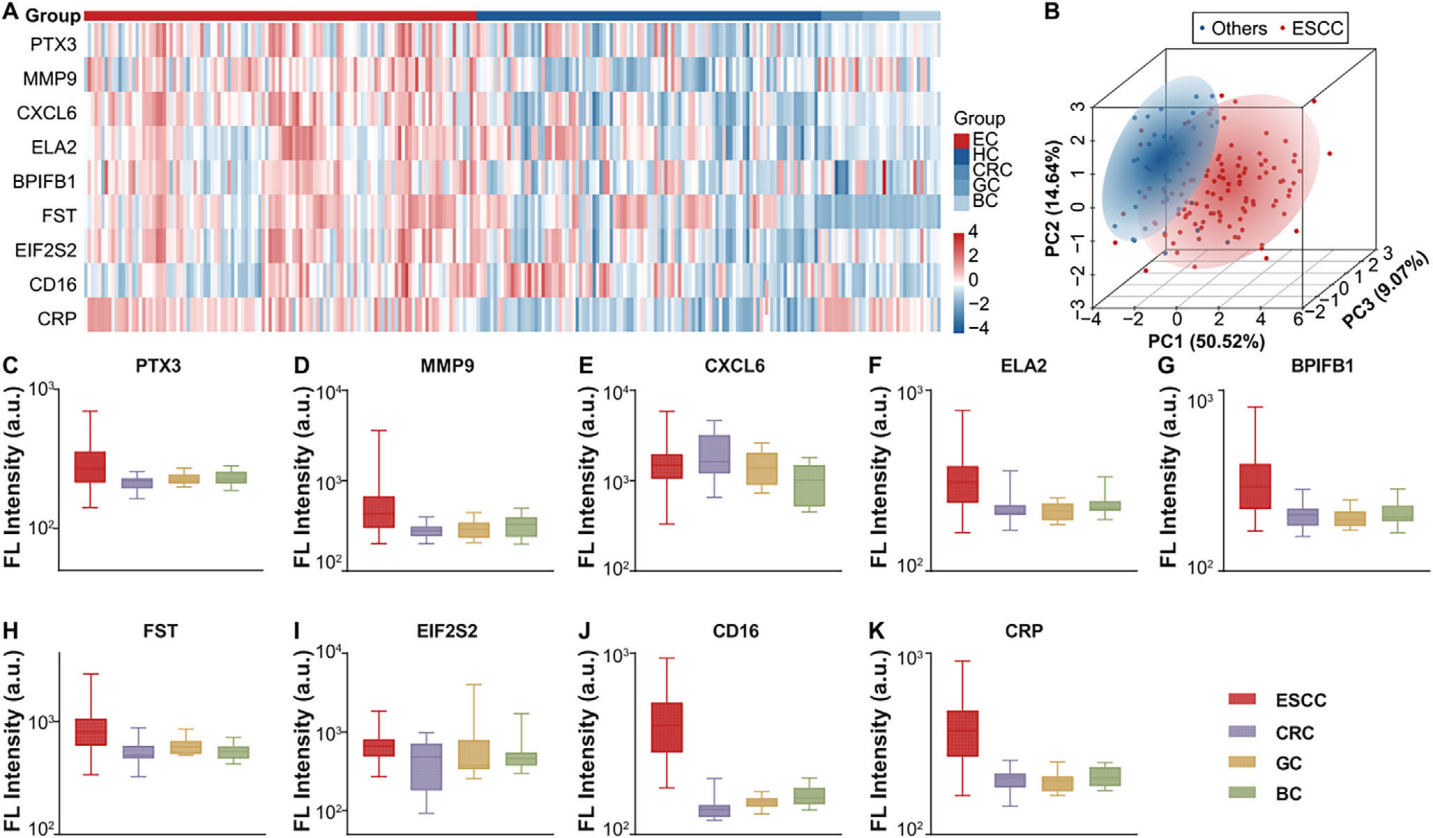
The specificity of ESCC small EVs proteins. Expression of ESCC biomarkers in other cancers. A) Heatmap showing the expression of 9 biomarkers in serum small EVs from HC and patients with ESCC, CRC, GC, and BC. B) PCA plot based on 9 biomarkers, comparing other cancers and ESCC. Each point represents a sample, with blue points indicating other cancers and red points representing samples from ESCC. The percentage values represent the explained variance. C) Box plot showing the average expression levels of 9 biomarkers in serum small EVs from patients with ESCC, CRC, GC, and BC. Abbreviations. BC: breast cancer; CRC: colorectal cancer; ESCC: esophageal squamous cell carcinoma; GC: gastric cancer; HC: healthy controls; PC: principal component; PCA: principal component analysis.

To evaluate the specificity of the 9‐DM model for ESCC diagnosis, serum samples were collected from patients with CRC, gastric cancer (GC), and breast cancer (BC). The gender and age distribution of patients is presented in Figure S24 (Supporting Information). In the serum small EVs of CRC, GC, and BC, the expression levels of the 9 markers show significant differences compared to those in ESCC (**Figure** **6**A; Figure S25, Supporting Information). The PCA based on 9‐DM suggests that ESCC samples exhibit distinct characteristics when compared to other cancer types (Figure 6B). However, PCA using the 9‐DM fails to effectively differentiate between CRC, GC, and BC samples in relation to HC samples (Figure S26A–C, Supporting Information). Notably, with the exception of CXCL6 and EIF2S2, the remaining seven markers were expressed at lower levels in the serum small EVs of CRC, GC, and BC patients (Figure 6C–K). Incorporating data from CRC, GC, and BC into the 9‐DM model completely failed to differentiate them from HC (Figure S26D,E, Supporting Information). These findings underscore the specificity of the selected small EVs protein markers and the 9‐DM model in diagnosing ESCC.

### 2.7 Tissue‐Based Validation of ESCC Biomarkers

To further validate the accuracy of our selected biomarkers, we performed proteomic analysis of EVs derived from ESCC cell lines (KYSE150/KYSE510) and normal esophageal epithelial cell lines (HEEC). The results demonstrated significant overexpression of PTX3, EIF2S2, and FST in ESCC‐derived EVs, with fold‐change values exceeding 10 compared to HEEC controls. Subsequent immunofluorescence analysis of three paired ESCC and adjacent normal tissue samples revealed tumor‐specific expression patterns, with positive signals in ESCC tissues but only faint or negligible staining in histologically normal adjacent regions (Figure S27, Supporting Information). These findings are consistent with previous reports documenting the tumor‐restricted expression profiles of these biomarkers.^[^ ^32^, ^33^, ^34^ ^]^

## 3 Discussions and Conclusion

In this work, although ESCC is taken as a model to test the application of the screened small EVs proteins panel to conduct early diagnosis, it is a universal method to screen the specific early‐diagnosis biomarkers, evaluate the biomarkers of treatment efficacy, and accurately prognosis biomarkers of other diseases. Early diagnosis can significantly improve patient prognosis.^[^ ^26^, ^35^ ^]^ Current endoscopic diagnostic and screening methods are limited, creating a need for new non‐invasive approaches to screen for and continuously monitor precancerous lesions.^[^ ^1^ ^]^ Although liquid biopsy has been explored in recent years, cfDNA has shown less than 20% sensitivity for the early diagnosis of EC.^[^ ^10^, ^11^, ^12^ ^]^ In contrast, small EVs are widely present in biological fluids and possess a double membrane structure, providing high stability.^[^ ^22^ ^]^ While several studies have demonstrated that the miRNAs in small EVs can differentiate tumor patients from healthy individuals, few have translated effectively into clinical applications.^[^ ^36^, ^37^ ^]^ Compared to RNA, small EVs‐derived proteins are more stable and therefore better suited for clinical examination.^[^ ^38^ ^]^ The increasing sophistication of proteomic assays in recent years has created an opportunity to understand protein function in both tumor patients and healthy individuals.^[^ ^39^, ^40^ ^]^

In this study, 4D‐DIA was used to identify and characterize all proteins in serum small EVs from patients and HC, revealing altered protein compositions in the small EVs of patients. Notably, important pathways such as small EVs composition, protein binding, and neutrophil degranulation are significantly upregulated, suggesting the functional status of their small EVs proteome. Additionally, pathways related to tumorigenesis, such as endocytosis, chemokines, and endothelial cell migration, are significantly elevated in the KEGG pathway analysis, indicating that small EVs proteins play a critical role in tumor formation and progression. This further supports the potential for identifying markers within the serum small EVs proteome for the diagnosis of cancer.

Due to the inherent heterogeneity of tumors and the complexity of their microenvironments,^[^ ^41^, ^42^ ^]^ it is challenging for a single marker to accurately reflect overall changes in tumor characteristics. Consequently, the combination of multiple markers has become a prevailing trend in diagnostic and therapeutic approaches.^[^ ^43^, ^44^ ^]^ This trend places higher demands on detection methods, emphasizing the need for assays with low sample consumption and high throughput capabilities, which are invaluable for marker detection and cancer diagnosis. CD63, CD81, and CD9 are transmembrane proteins present in the small EVs membrane that represent incompletely overlapping small EVs populations.^[^ ^22^, ^24^, ^25^ ^]^ The microfluidic platform developed in this study utilizes a mixture of antibodies against CD63, CD9, and CD81 to specifically capture serum small EVs, achieving a capture efficiency of over 90% at concentrations of 10^8^ to 10^10^ particles mL^−1^. The captured small EVs undergo in situ cleavage on the chip, yielding total small EVs proteins, including both membrane and internal proteins. This approach minimizes the loss of total small EVs proteins and enhances the overall sensitivity of the assay. The entire process requires only 10 µL of serum sample volume, significantly reducing sample loss. Furthermore, the barcode immunoassy platform enables the simultaneous detection of at least 14 markers across 60 clinical samples, significantly reducing the time required for multi‐sample analysis. This makes it particularly well‐suited for screening a diverse population. Notably, the detection limit of the chip for proteins is as low as 0.01–96.61 pg mL^−1^, and fluorescence readings are relatively stable. Thus, the barcode immunoassy platform developed in this study holds substantial clinical application potential for the detection of small EVs proteins.

While serum small EVs proteomics are suited to characterize proteins in diseases and identify promising biomarkers for diagnosis, interpreting complex histological data remains a challenge.^[^ ^16^, ^45^, ^46^, ^47^ ^]^ Machine learning is increasingly recognized as a valuable tool for enhancing the accuracy of medical monitoring and diagnosis. In this study, the random forest algorithm was utilized to select the optimal model by adjusting parameters based on ACC and the AUC. The accuracy of the diagnostic model reaches 79.1% in test set 1. To further improve accuracy and reduce detection costs, the best model, the 9‐DM, is identified through machine learning, achieving an accuracy of 88.4% in test set 1. Additionally, machine learning algorithms reveal the predictive potential of certain small EVs proteins that are often overlooked by traditional analytical methods. For instance, although CD16 and ELA2 did not exhibit significant differences when comparing the overall mean values between ESCC patients and HC, these two markers were retained in the model's selection of optimal variables. The machine learning algorithms used to create the diagnostic models were validated for generalization and demonstrated superior performance compared to clinically available squamous cancer markers. Specifically, Cohort 2 was validated as an independent external test set, where the 14‐DM and 9‐DM models achieved accuracies of 82.1% and 91.0%, respectively. Notably, the 9‐DM model maintained robust performance in the subsequent Cohort 3 (83.3% accuracy; AUC = 0.93), thereby confirming its generalizability across distinct patient populations. Furthermore, the 9‐DM model demonstrates strong performance in identifying early‐stage ESCC patients. In a cohort of stage I‐II ESCC patients and HC, the model accurately identified 85.7% of early ESCC patients, resulting in an overall accuracy of 90.8%, which surpasses existing clinical markers. Molecular characterization revealed significant overexpression of PTX3, FST, and EIF2S2 in ESCC‐derived extracellular vesicles (FC > 10), with tumor‐specific expression patterns confirmed by immunofluorescence. These findings collectively validate the model's clinical utility while elucidating molecular foundations, supporting its further development through expanded validation studies and algorithmic refinement for clinical implementation.

Additionally, serum samples from patients with CRC, GC, and BC were tested in this study. Compared to ESCC, the proteins identified in these three cancer types generally exhibit lower expression levels. The 9‐DM model can accurately identify ESCC patients. This further substantiates the specificity of the selected markers and the diagnostic model for ESCC. Overall, our strengths lie in performing a proteomic analysis of serum small EVs and describing the functional landscape of serum small EVs proteins in patients. A set of straightforward protein markers has been screened, and a machine learning approach has been applied to construct a robust diagnostic model for ESCC, facilitating replication, optimization, and clinical application. Additionally, a barcode immunoassay platform has been developed that can utilize 10 µL serum samples for simultaneous high‐throughput assays involving multiple samples and parameters, which holds significant translational value for clinical applications.

This study has several limitations that should be acknowledged. First, as our predictive model was developed using relatively quantitative proteomics data, its clinical application for ESCC risk assessment in new patients will require standardized quality control samples during model implementation. Despite these limitations, our work has successfully identified key EVs proteome markers that effectively discriminate ESCC from healthy controls, representing a crucial advancement toward clinically applicable diagnostic models. Second, the current platform requires manual data transfer of test results to the machine learning model, which may introduce operational complexity in clinical settings. Moving forward, we will pursue absolute quantitative proteomic analysis of these biomarkers in large‐scale multicenter cohorts to establish clinically relevant reference ranges and detection thresholds, while simultaneously working to develop a fully integrated automated platform that combines detection, data acquisition, and diagnostic output to enhance both clinical utility and operational efficiency.

In conclusion, the proposed platform identifies biomarkers with potential diagnostic value and constructs a diagnostic model incorporating machine learning algorithms. Furthermore, a barcode immunoassay platform has been developed that integrates small EVs capture, their in situ lysis, and multiparameter detection, enabling the rapid analysis of multiple samples. The understanding of disease pathology is enhanced through these findings, which also facilitates early detection and develops new methods for multi‐parameter small EVs detection. More broadly, the machine learning‐based interpretation of small EVs data offers distinct advantages in tumor detection and clinical decision‐making, while the developed microfluidic assay platform holds significant translational prospects for practical clinical applications and may be extended to the study of other diseases.

## 4 Experimental Section

### Clinical Cohort

Between August 2023 and May 2024, 66 patients with ESCC were recruited from Qilu Hospital of Shandong University, and 80 HC who underwent physical examinations were included, forming Cohort 1. Between July 2023 and September 2023, an additional 47 ESCC patients, treated at the Provincial Hospital of Shandong First Medical University, and 20 HC, were recruited, thus establishing Cohort 2. Furthermore, from January to March 2025, 30 ESCC patients and 30 HC were enrolled at Qilu Hospital to establish Cohort 3 for validation studies (Figure S28, Supporting Information). Inclusion criteria: Participants were aged 18–85 years, had histologically confirmed ESCC, and were untreated. Exclusion criteria: Individuals with dual or multiple primary tumors; participants with immune disorders or autoimmune diseases; and those who had undergone organ transplantation, non‐autologous bone marrow transplantation, or stem cell transplantation were excluded. Inclusion criteria for healthy individuals: 1) Written consent obtained; 2) Age ranged from 18 to 85 years; and 3) Absence of malignant disease. The exclusion criteria mentioned above also applied to healthy individuals. All samples used in this study were retrospectively obtained from established institutional biobanks. The use of human materials was approved by the Medical Science Research Ethics Committee of Qilu Hospital, Shandong University (Approval Nos. KYLL‐2021(KS)‐011 and KYLL‐202409(YJ)‐024). All participants voluntarily signed the informed consent form.

### Collection of Clinical Samples

Serum samples were collected from ESCC patients and HC. A 5 mL peripheral venous blood sample was obtained from each participant. The samples were promptly transferred to the laboratory within 1 h at 4 °C for further processing. Freshly collected blood samples were kept at room temperature (RT) for 1 h to allow clotting before centrifugation. The supernatant was then carefully removed by centrifugation at 4 °C and 1,000 g for 15 min and transferred to a clean test tube. To ensure complete removal of platelets and other debris, the samples underwent a second centrifugation at 4 °C and 10 000 g for 10 min. And the collected supernatant was stored at −80 °C.

### Collection and Isolation of Cell Supernatants Small EVs

Cells were cultured in complete medium until reaching 70% confluence, at which point the medium was exchanged under fetal bovine serum (FBS)‐free medium. Supernatants were collected after 48 h of culture in FBS‐free conditions. The collected supernatants underwent serial centrifugation: first at 300 × g for 10 min, followed by 2000 × g for 10 min, and then at 10 000 × g for 30 min. Then the supernatants were passed through a 0.22‐µm filter and subjected to ultracentrifugation at 100 000 × g for 70 min. The supernatant obtained after centrifugation was designated as the small EVs‐removed fraction. The small EVs pellets were then resuspended in phosphate buffer saline (PBS) and underwent a second ultracentrifugation at 100 000 × g for 70 min to purify the small EVs.

### Nanoparticle Tracking Analysis

Small EVs were resuspended and diluted 100‐ to 500‐fold to obtain a concentration of 20–100 particles per frame. Analysis was performed using the NanoSight NS300 system (NanoSight Technology, Malvern, UK), which features a 488 nm laser and a highly sensitive sCMOS camera. Following the manual introduction of small EVs into the chamber, each sample was measured in triplicate using a 13‐stage camera, with a 30‐s acquisition time and a detection threshold set to 7. NTA analysis software version 2.3 was used to analyze a minimum of 200 complete traces per video.

### Transmission Electron Microscopy

Small EVs particles were treated with 2.5% glutaraldehyde for 10 min at RT. A drop of 5–10 µL of the small EVs’ suspension was then placed on a copper grid. Excess liquid was removed using filter paper, and the small EVs samples were washed three times with PBS, with each wash lasting 10 min. Finally, 10 µL of 1% uranyl acetate was applied for 1 min to negatively stain the small EVs on the copper mesh, which were then examined using a Tecnai G2 F20 transmission electron microscope.

### Serum Small EVs for Liquid Chromatography‐Mass Spectrometry (LC‐MS) and Data Independent Acquisition

Serum small EVs were isolated and collected using the EVtrap kit. After isolation, the small EVs underwent lysis, protein extraction, reduction, alkylation, and trypsin digestion to generate peptide chains for mass spectrometry analysis. Next, peptides from each sample were subjected to mass spectrometry for DIA analysis. Additional details can be found in Figure S29 (Supporting Information).

### Differential Protein and Pathway Enrichment Analysis

Differential protein abundance between ESCC patients and HC was calculated using the “limma” package in R software. The significance level was set to an adjusted *P*‐value of < 0.05, with a log2 fold change (FC) threshold of > 0.58 (indicating upregulation) or < −0.58 (indicating downregulation in the tumor). Functional enrichment analysis was performed using KOBAS (http://bioinfo.org/kobas) with default parameters.

### Fabrication of Microfluidic Platforms

GOQDs substrate glass slides and PDMS microchamber layers were prepared following the same procedure previously reported.^[^ ^31^ ^]^ The microprinting chip with 14 parallel micorchannels was first bonded to GOQD‐functionalized glass slides, with this nanomaterial substrate demonstrating significantly enhanced antibody immobilization efficiency. Using a precision pipette, 3 µL of capture antibody solution was dispensed into each inlet of the independent microchannel, followed by a vacuum force to pump the loaded capture antibody from the inlet to the outlet at room temperature. The capture antibody was immobilized on the substrate surface in each corresponding microchannel. After the microprinting chip was removed, the substrate was treated with 1% BSA solution for surface passivation, followed by blocking with 3% BSA solution to saturate any remaining reactive sites outside the microchannels. Finally, the chips underwent rigorous washing, spin‐drying, and storage at 4 °C until use. The microprinting chip contained 14 zigzag‐patterned microchannels that maintain complete fluidic isolation. Each detection unit included a full circulation loop of all 14 microchannels (Figure S30, Supporting Information). To capture small EVs, 4.5 µL of a mixed solution containing antibodies of CD63 (100 µg mL^−1^), CD81 (100 µg mL), and CD9 (100 µg mL^−1^) was added to each reaction unit and incubated the mixture overnight. Blocking was then performed using 3% BSA for 10 min, followed by successive washes with PBS and ultrapure water to remove unbound components. To enrich small EVs, 10 µL of serum sample was added to each reaction unit and incubated for 60 min. The supernatant was then removed, and the wells were rinsed with PBS. Subsequently, 5 µL of a mixture of RIPA lysis buffer and protease inhibitor (in a 100:1 ratio) was added to each reaction unit to lyse the captured small EVs in situ for 30 min. For antigen detection, the PDMS microchamber of the microarray chip was sealed with 1% BSA. The small EVs lysate was then transferred to the wells on the microarray chip and incubated for 45 min, after which the supernatant was removed. The microarray chip was incorporated into 1% BSA, and the wells were incubated with a detection antibody at a final concentration of 5 µg mL^−1^ for 45 min, followed by incubation with APC for 30 min. The microarrays were thoroughly washed with PBS and distilled water and then shaken dry. The fluorescence intensity of the antigen was measured using a fluorescence scanner. The commercial antibodies and proteins used in this study were sourced from manufacturers such as R&D Systems, BioLegend, NOVUS, eBioscience, Abcam, and Santa Cruz Biotechnology.

### Microfluidic Platforms Data Analysis

The detection process involves the following steps: 1) Signal Acquisition: The biochip was scanned using a 635 nm laser channel, and fluorescence signals from each barcode were quantified using GenePix Pro software, and the average fluorescence intensity from the detection area was achieved. 2) Data Normalization: Raw fluorescence intensity values were log10‐transformed prior to machine learning model input to normalize data distribution and enhance model performance.

### Diagnostic Predictive Modeling

A diagnostic prediction model was developed for ESCC using R version 4.4.2, employing an integrated machine learning approach for multidimensional analysis. Methodologically, multiple R packages were systematically implemented: randomForest for random forest modeling, caret for data preprocessing and model tuning, pROC for performance evaluation, randomForestExplainer for feature importance analysis, ggplot2 for visualization, and Rtsne for high‐dimensional data dimensionality reduction. During model construction: data standardization and stratified sampling (70% training set/30% test set) were first performed using the caret package to ensure balanced data distribution. The random forest model was developed using the bootstrap aggregation algorithm implemented in randomForest, initially incorporating 14 plasma proteins as predictive variables. Through caret‐guided grid search validation, the model parameters were optimized to: ntree = 145 decision trees, mtry = 14 (number of features considered at each node split), and nodesize = 5 (minimum samples per terminal node). Parameter optimization was guided by both AUC values calculated by pROC and confusion matrix metrics generated by caret. Feature importance analysis was conducted using randomForestExplainer, which provided three complementary evaluation methods: 1) minimum depth analysis (min_depth_distribution) to identify the most classification‐critical features; 2) node purity improvement (measure_importance) to quantify each feature's contribution to model accuracy; and 3) root node splitting frequency to reflect global feature importance. Based on these analyses, the 9 most predictive protein biomarkers were selected from the initial 14 features for the final model. The validation phase employed a multidimensional evaluation strategy: the caret‐generated confusion matrix provided key metrics including accuracy, sensitivity, and specificity; pROC calculated AUC values from ROC curves; ggplot2 produced calibration curves; and Rtsne visualization demonstrated the separation pattern between ESCC patients and healthy controls in the protein biomarker space. The model outputs individualized prediction probabilities (range 0–1) through randomForest's predict function, with a diagnostic threshold set at 0.5 (probability > 0.5 classified as ESCC, ≤0.5 as healthy controls). All visualizations were created using ggplot2, including ROC curves, multi‐dimensional feature importance heatmaps, t‐SNE dimensionality reduction scatter plots, and model calibration curves.

### Immunofluorescence Staining

Formalin‐fixed, paraffin‐embedded sections were deparaffinized through xylene and graded ethanol series, followed by antigen retrieval in citrate buffer (pH 6.0) using microwave heating. After blocking with 10% serum matching the secondary antibody host species, slides were incubated with the following primary antibodies at 4 °C overnight: anti‐FST (1:400, Proteintech #60060‐1‐Ig), anti‐PTX3 (1:200, Proteintech #13797‐1‐AP), and anti‐EIF2S2 (1:500, Servicebio #GB111135). Alexa Fluor‐conjugated secondary antibodies (1:200) were applied for 50 min at room temperature, followed by DAPI counterstaining and autofluorescence quenching. Sections were mounted with anti‐fade medium and imaged using a digital slide scanner. PBS washes (3 min × 5 min) were performed between all steps.

### Statistical Methods

The Kolmogorov–Smirnov test was applied to assess the normality of all data, while categorical data were analyzed using the chi‐square test. And for data that were normally distributed, unpaired two‐tailed *t*‐tests were utilized for comparisons, while the Mann–Whitney nonparametric test was used for non‐normally distributed data. The linear relationship between fluorescence intensity or optical density (O.D.) values and target antigen concentration was evaluated using the R‐squared (R^2^) statistic. A *p*‐value of below 0.05 was considered indicative of statistical significance. The limit of detection (LOD) was determined by the equation: LOD = 3 × δ /S, where 𝛿 is the relative standard deviation of the blank value, and 𝑆 denotes the slope derived from the linear regression equation. Diagnostic efficiency was assessed using accuracy, ROC curves, and other relevant metrics. Analyses were conducted using GraphPad Prism (v.9.5), R software (v.4.4.1) (available at https://www.r‐project.org/), and Origin (v.2021).

## Conflict of Interest

The authors declare no conflict of interest.

## Author Contributions

L.H., Y.Z., J.W., X.Z., and C.M. conceived the idea of the study. X.Z., Y.Z., J.Q., and Y.L. performed the experiments, article writing, and created the figures and tables. Y.Z., C.W., M.S., and X.C. guided the preparation of this manuscript. Z.L. and Y.Z. performed the sample collection and data acquisition. H.L., L.H., and C.M. conducted paper revisions. All authors read and approved the final manuscript.

## Supporting information



Supporting Information

## Data Availability

The data that support the findings of this study are available from the corresponding author upon reasonable request.
