# Supplementary material for: Microfluidic Biochip‐Based Multiplexed Profiling of Small Extracellular Vesicles Proteins Integrated with Machine Learning for Early Disease Diagnosis
Source: Adv Sci (Weinh). 2025 Jul 7;12(37):e06167. doi: 10.1002/advs.202506167 (PMC12499472; doi:10.1002/advs.202506167)
Supplement: Supplementary file 1 — Supporting Information [file ADVS-12-e06167-s001.pdf]

ADVANCED SCIENCE
Open Access

Supporting Information

for *Adv. Sci.*, DOI 10.1002/advs.202506167

Microfluidic Biochip-Based Multiplexed Profiling of Small Extracellular Vesicles Proteins Integrated with Machine Learning for Early Disease Diagnosis

Xue Zhang, Yibin Jia, Zhikai Li, Yunhong Zhang, Chao Wang, Yanbo Liang, Jiaoyan Qiu, Mingyuan Sun, Xiaoshuang Chen, Miao Huang, Yu Zhang, Jianbo Wang, Hong Liu, Chuanbin Mao* and Lin Han**

Supplementary Materials

Microfluidic Biochip-Based Multiplexed Profiling of Small Extracellular Vesicles Proteins Integrated with Machine Learning for Early Disease Diagnosis

Xue Zhang[a], Yibin Jia[b], Zhikai Li[b], Yunhong Zhang[a], Chao Wang[a], Yanbo liang[a], Jiaoyan Qiu[a], Mingyuan Sun[a], Xiaoshuang Chen[a], Miao Huang[a], Yu Zhang[a], Jianbo Wang[b*], Hong Liu[a], Chuanbin Mao[c*], Lin Han[a,d*]

[a]Institute of Marine Science and Technology, Shandong University, Qingdao, China.

[b]Department of Radiation Oncology, Qilu Hospital, Cheeloo College of Medicine, Shandong University, Jinan, China.

[c] Department of Biomedical Engineering, The Chinese University of Hong Kong, Sha Tin, Hong Kong SAR, China

[d]State Key Laboratory of Microbial Technology, Shandong University, Tsingdao, China.

Contents

Figure. S1. Bar chart displaying the expression levels of 14 proteins detected by mass spectrometry. 4
Figure. S2. Box plot displaying the average expression levels of 14 proteins detected by mass spectrometry. 5
Figure. S3. Characterization of small EVs. 6
Figure. S4. Optimization of chip conditions and microarray presentation. 7
Figure. S5. Extracellular vesicles capture capacity of the microfluidic chip platform..8
Figure. S6. Comparative analysis of biomarker signals in EVs-capture chips versus antibody-free control chips using three representative patient samples.. 9
Figure. S7. Long-term stability assessment of the biochip platform.. 10
Figure. S8. Standard curves for the detection of 14 proteins on the barcode chip. 11
Figure. S9. Specificity of the detection of 14 proteins on the barcode chip. 12
Figure S10. Bar chart of the fluorescence values for the detection of 14 proteins in ESCC and HC in mass spectrometry cohort. 13
Figure S11. Box plot comparing the mean expression levels of 14 proteins in ESCC and HC in mass spectrometry cohort.. 14
Figure S12. Information on the clinical cohort. 15
Figure S13. Bar chart of the fluorescence values for the detection of 14 proteins in cohort 1.. 16
Figure S14. Box plot comparing the mean expression levels of 14 proteins in ESCC and HC in cohort 1. 17
Figure S15. Bar chart of the fluorescence values for the detection of 14 proteins in cohort 2. 18
Figure S16. Box plot comparing the mean expression levels of 14 proteins in ESCC and HC in cohort 2. 19
Figure S17. Detection of SCC. 20
Figure S18. ROC assessment of 14 biomarkers. 21
Figure S19. Parameter selection and results presentation for the construction of the

14-DM model. 22
Figure S20. Parameter selection and results presentation for the construction of the 9-DM model. 23
Figure S21. Bar chart of the fluorescence values for the detection of 14 proteins in cohort 3 24
Figure S22. Box plot comparing the mean expression levels of 14 proteins in ESCC and HC in cohort 3. 25
Figure S23. 9-DM model in cohort 3. 26
Figure S24. Basic information on samples from other cancer types. 27
Figure S25. Bar chart displaying the fluorescence values of detections including other cancer types. 28
Figure S26. Presentation of machine learning results for other cancer types. 29
Figure S27. Comparative immunofluorescence analysis of candidate biomarkers. ... 30
Figure S28. Flowchart of cohort screening and construction. 31
Figure S29. Schematic of the workflow for 4D-DIA mass spectrometry analysis of serum small EVs. 32
Figure S30. Micorchannels structure of the microprinting chip. 34
Table S1. Comparison of microfluidic biochips for the proteins detection. 35
Table S2. Baseline characteristics of esophageal squamous cell carcinoma patients in cohort 1, cohort 2 and cohort 3. 37
References.. 38

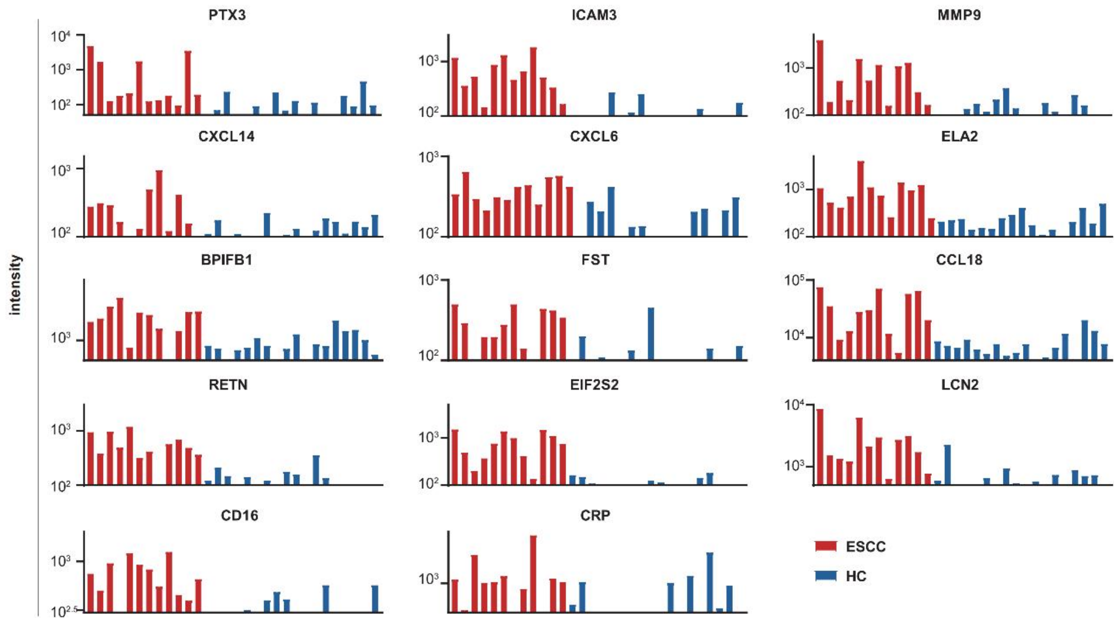

Figure S1. Bar chart displaying the expression levels of 14 proteins detected by mass spectrometry.

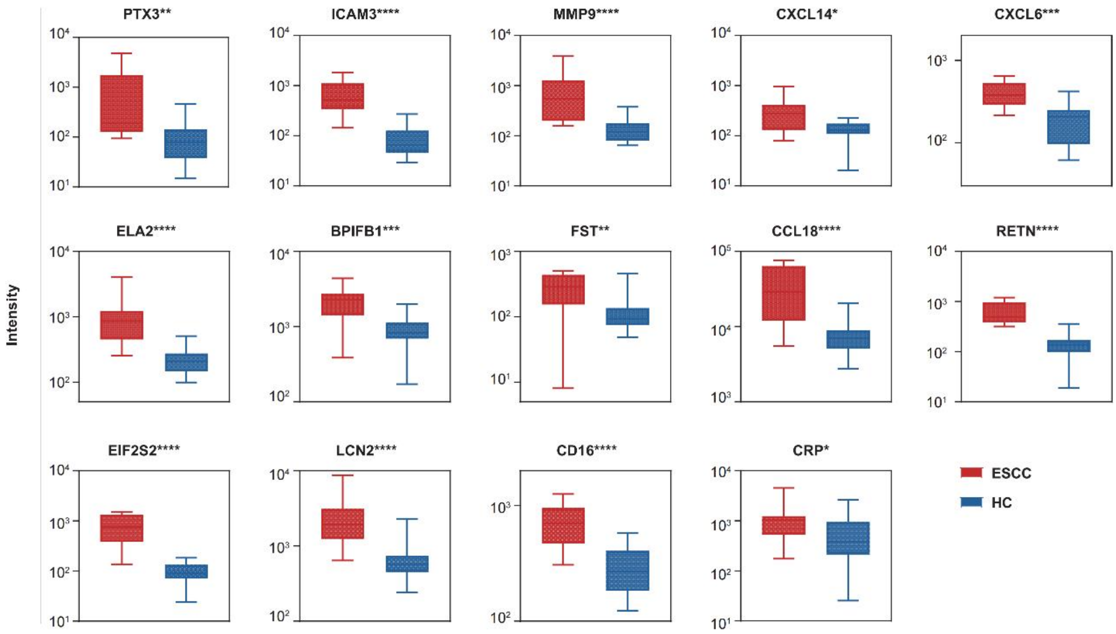

Figure S2. Box plot displaying the average expression levels of 14 proteins detected by mass spectrometry.

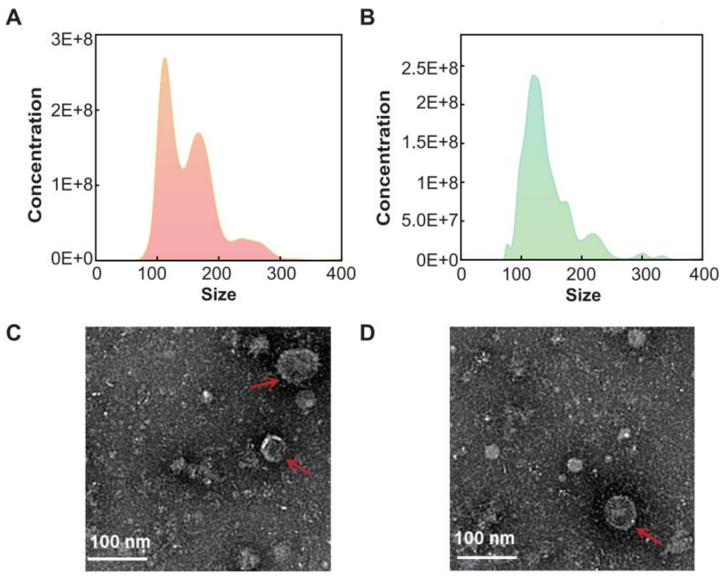

Figure S3. Characterization of small EVs. A. NTA characterization of KYSE510 cells and B. KYSE150 cells. C.TEM characterization of KYSE510 cells and D. KYSE150 cells.

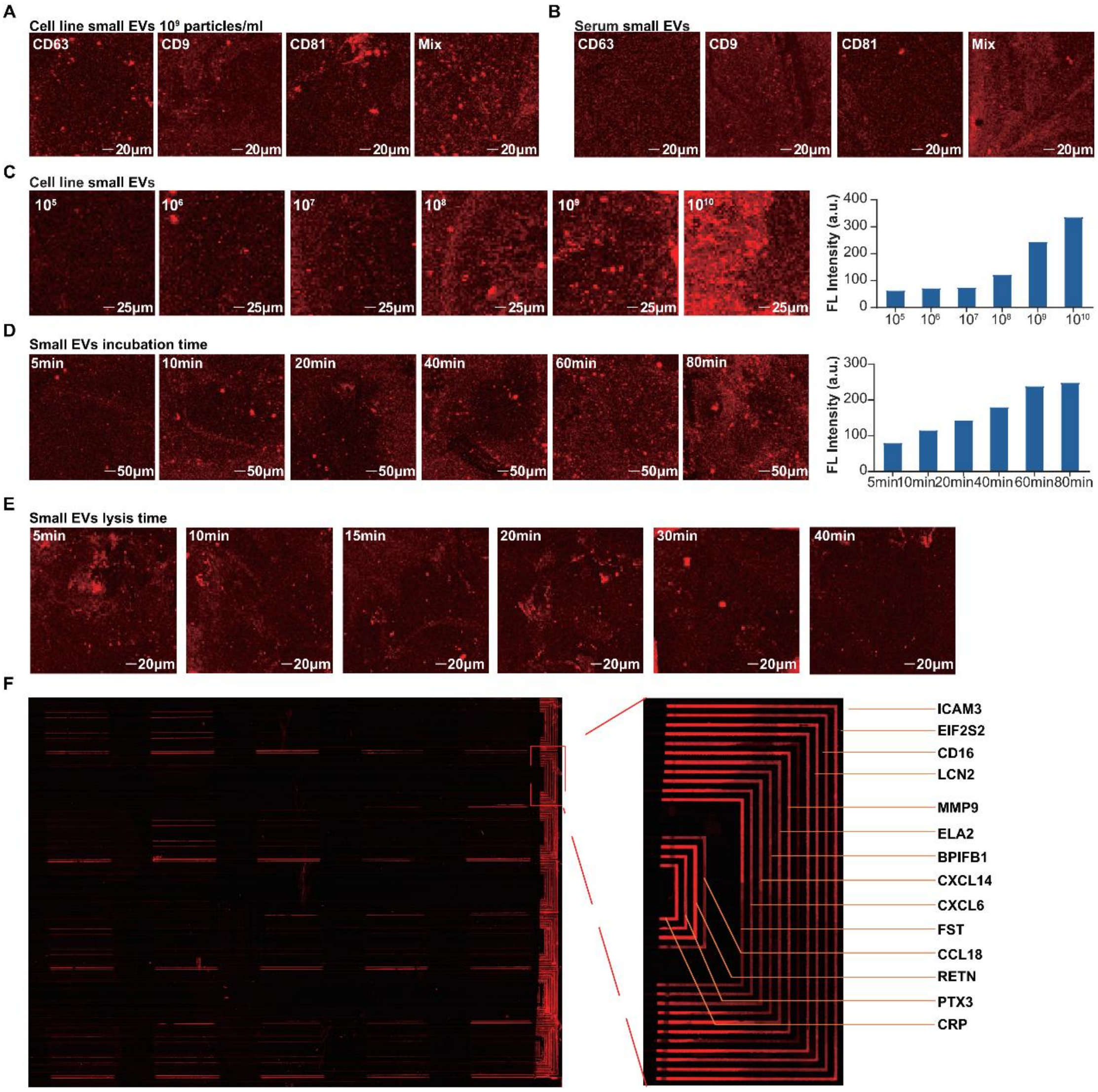

Figure S4. Optimization of chip conditions and microarray presentation. A. Capture of small EVs by different antibodies from the KYSE150 cell line and B. ESCC serum. C. Capture of small EVs from cell lines at different concentrations using the chip with immobilized mixed antibodies. D. Capture of small EVs at a concentration of 10^9 particles/mL on the chip during incubation at different time points. E. Residual small EVs at a concentration of 10^9 particles/mL on the chip after different lysis times. F. Microarray fluorescence scan image of the barcode chip detection.

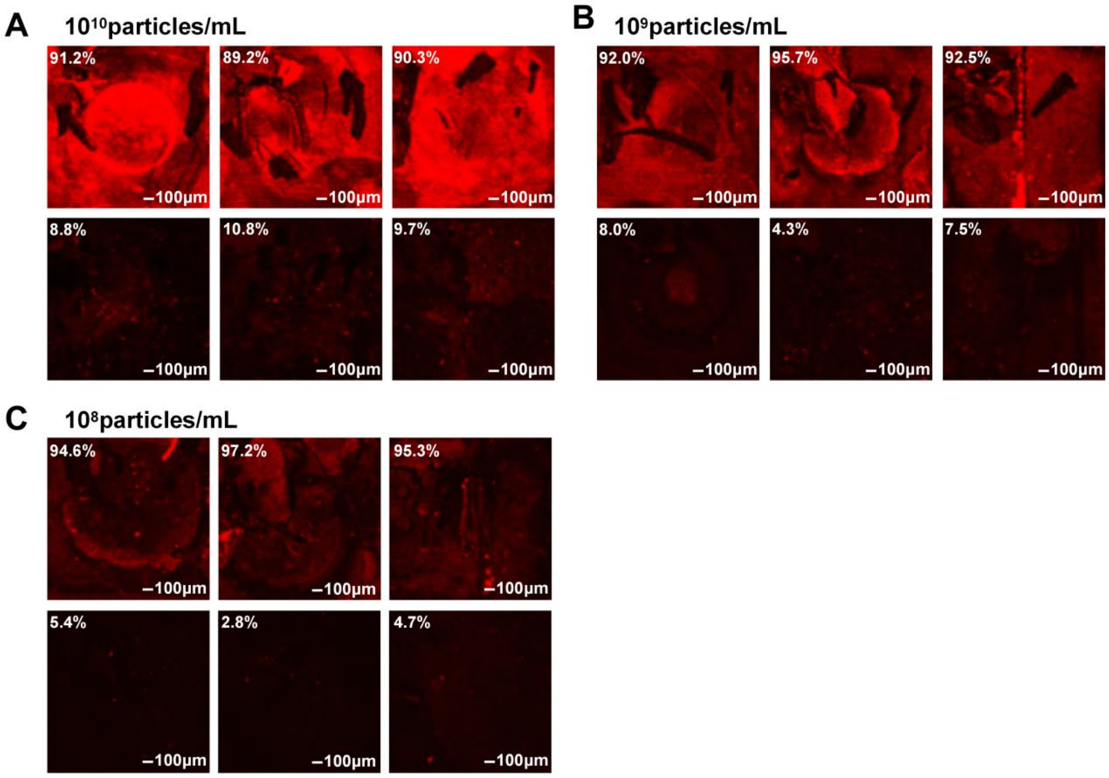

Figure S5. Extracellular vesicles capture capacity of the microfluidic chip platform. Figures A, B, and C show EVs capture at concentrations of 10^8, 10^9, and 10^{10} particles/mL, respectively. Captured small EVs are detected using CD63 antibodies conjugated with APC, and scanning is performed with a fluorescence scanner with a 635 nm laser source. Calculation formula: [Initial fluorescence intensity/(Initial fluorescence intensity + Recovered fluorescence intensity)] ×100 %.

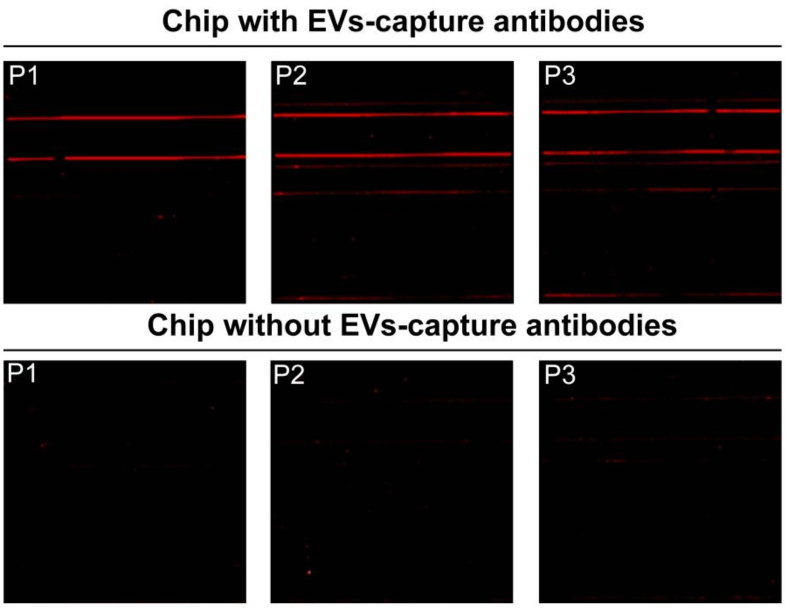

Figure S6. Comparative analysis of biomarker signals in EVs-capture chips versus antibody-free control chips using three representative patient samples.

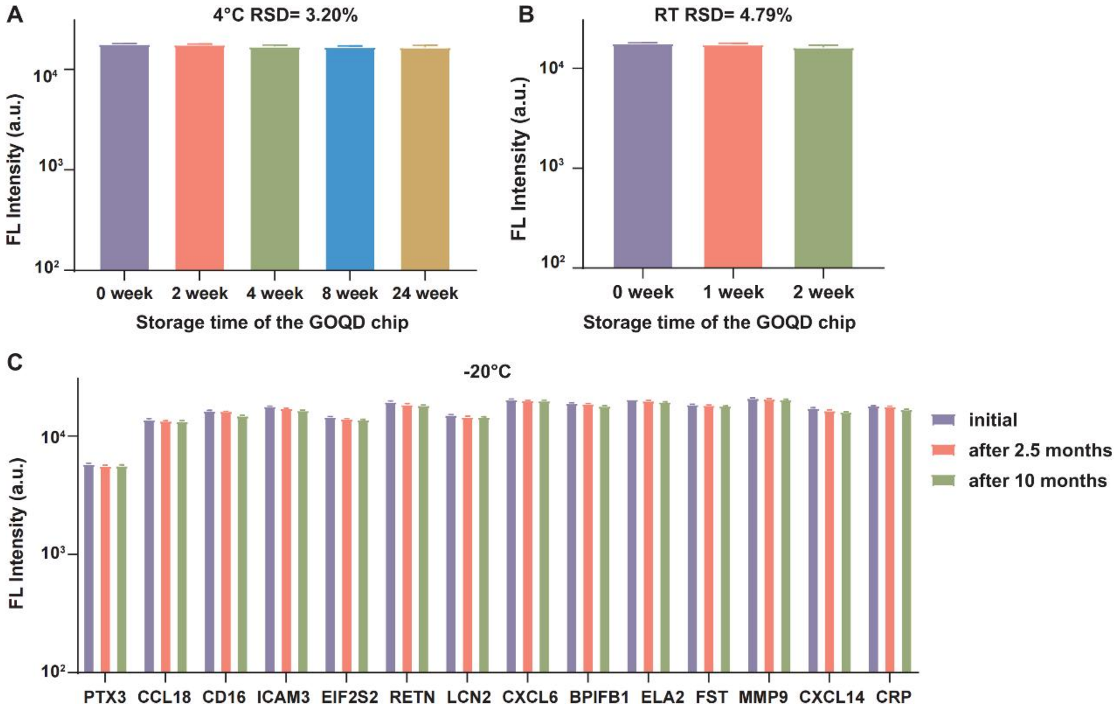

Figure S7. Long-term stability assessment of the biochip platform. A-B. Stability evaluation of GOQD-functionalized slides stored at 4°C for 24 weeks and at room temperature for 2 weeks. Following antibody immobilization on substrates, fluorescence intensity was quantified using fluorophore-conjugated IgG detection (488 nm excitation). C. Stability assessment of antibody-conjugated chips stored at -20°C for 2.5 and 10 months. Preserved chips with immobilized fluorophore-labeled IgG antibodies were rescanned (488 nm excitation) to determine signal retention.

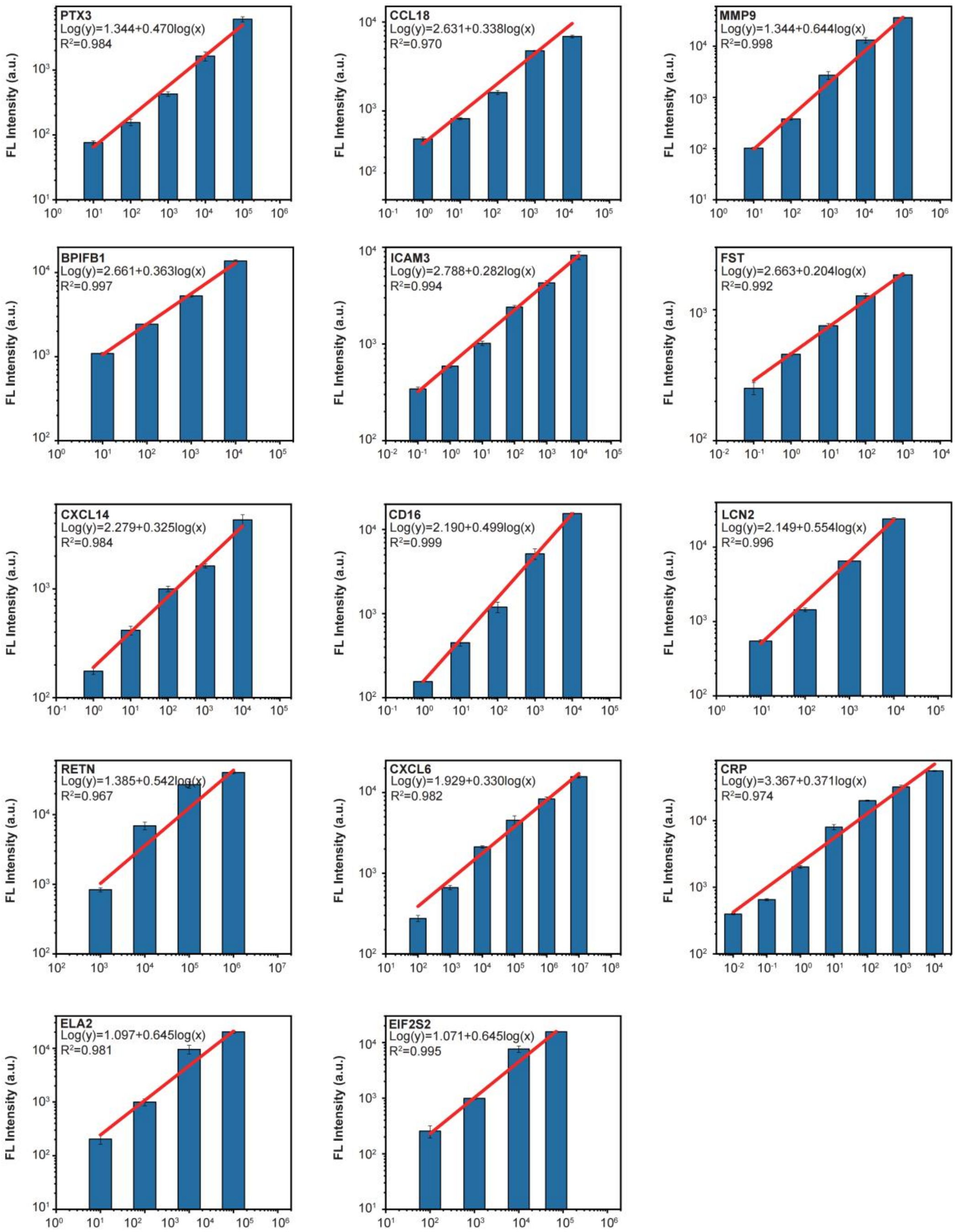

Figure S8. Standard curves for the detection of 14 proteins on the barcode chip. Standard curves were established by correlating fluorescence intensities with recombinant protein concentrations serially diluted in 1% BSA, demonstrating linear detection across at least four orders of magnitude.

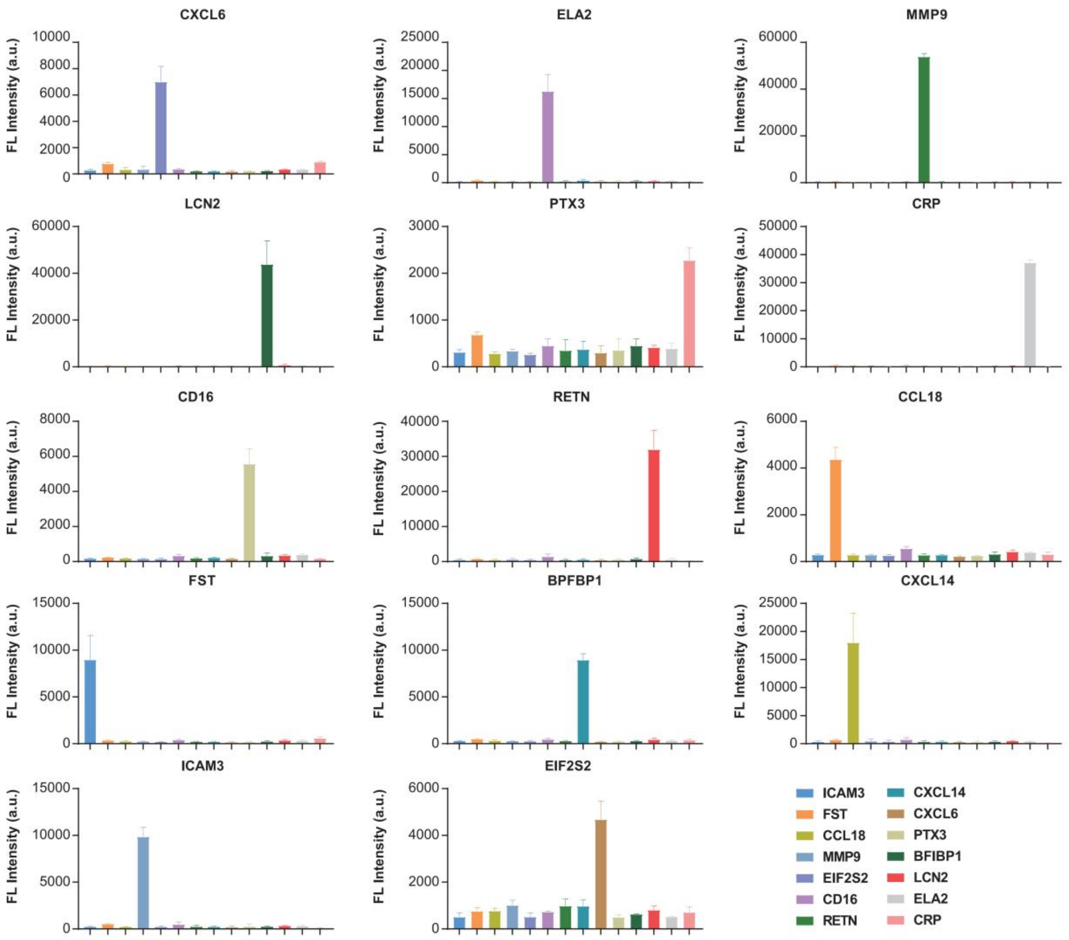

Figure S9. Specificity of the detection of 14 proteins on the barcode chip.

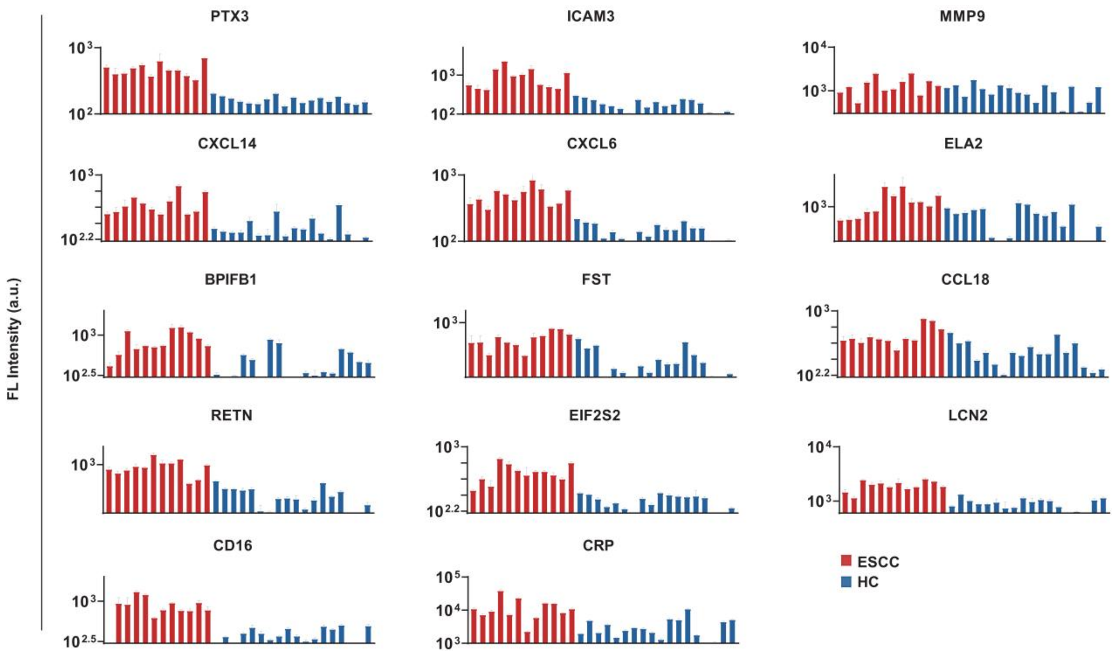

Figure S10. Bar chart of the fluorescence values for the detection of 14 proteins in ESCC and HC in mass spectrometry cohort.

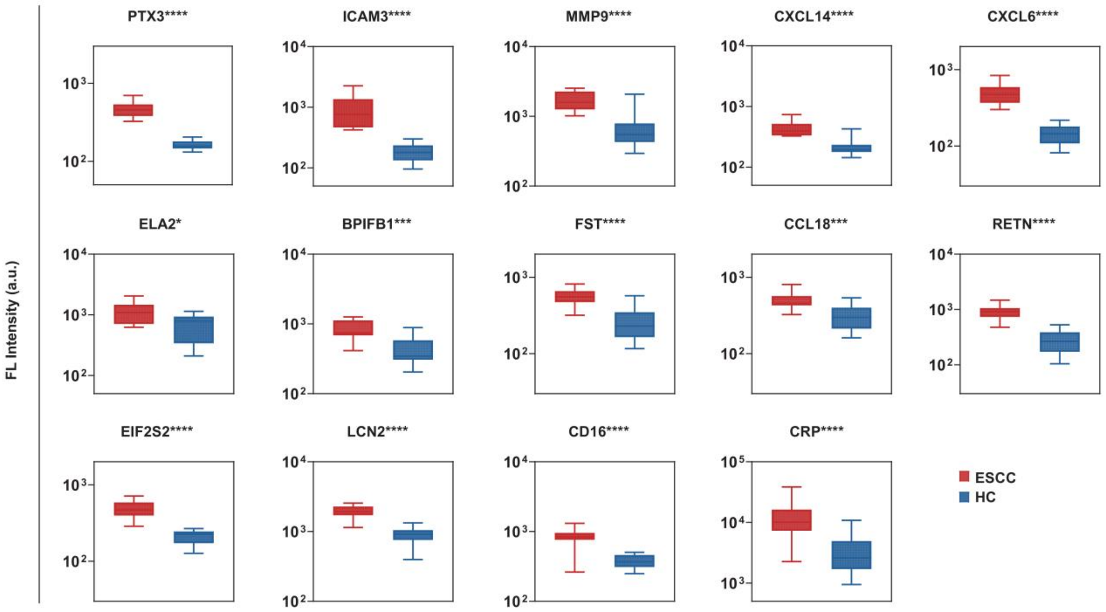

Figure S11. Box plot comparing the mean expression levels of 14 proteins in ESCC and HC in mass spectrometry cohort.

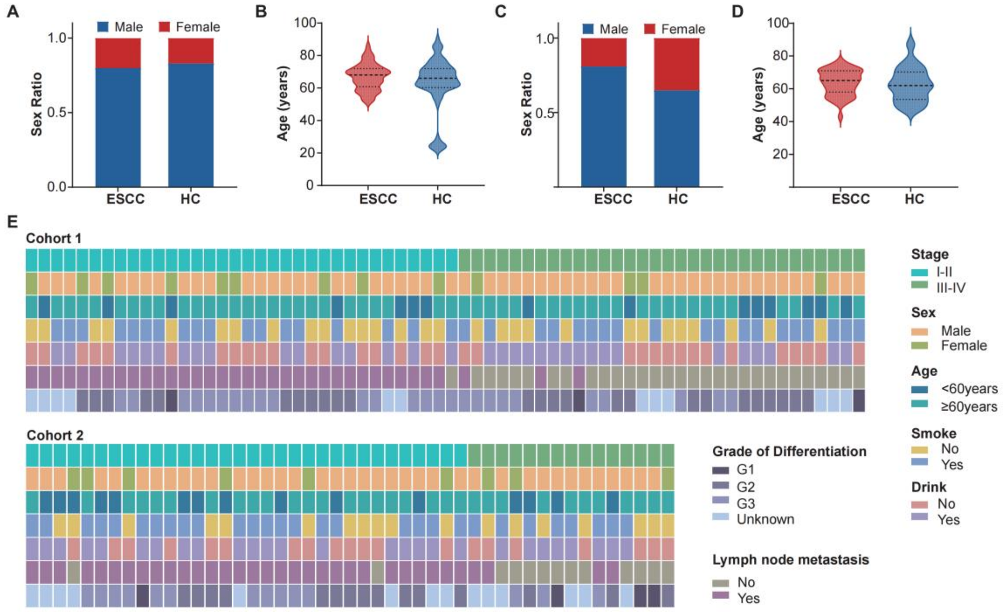

Figure S12. Information on the clinical cohort. A. Bar chart displaying the gender ratio of ESCC and HC in cohort 1. B. Violin plot showing the age distribution of ESCC and HC in cohort 1. C. Bar chart displaying the gender ratio of ESCC and HC in cohort 2. D. Violin plot showing the age distribution of ESCC and HC in cohort 1. E. Heatmap displaying the clinical pathological information of esophageal cancer patients in cohort 1 and cohort 2.

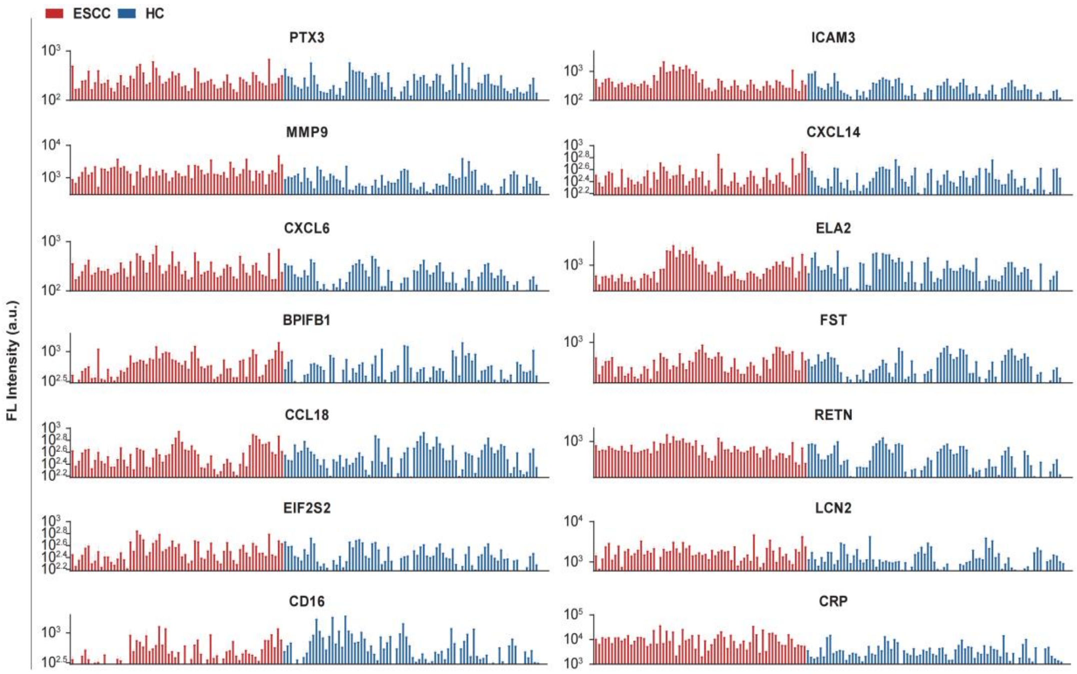

Figure S13. Bar chart of the fluorescence values for the detection of 14 proteins in cohort 1.

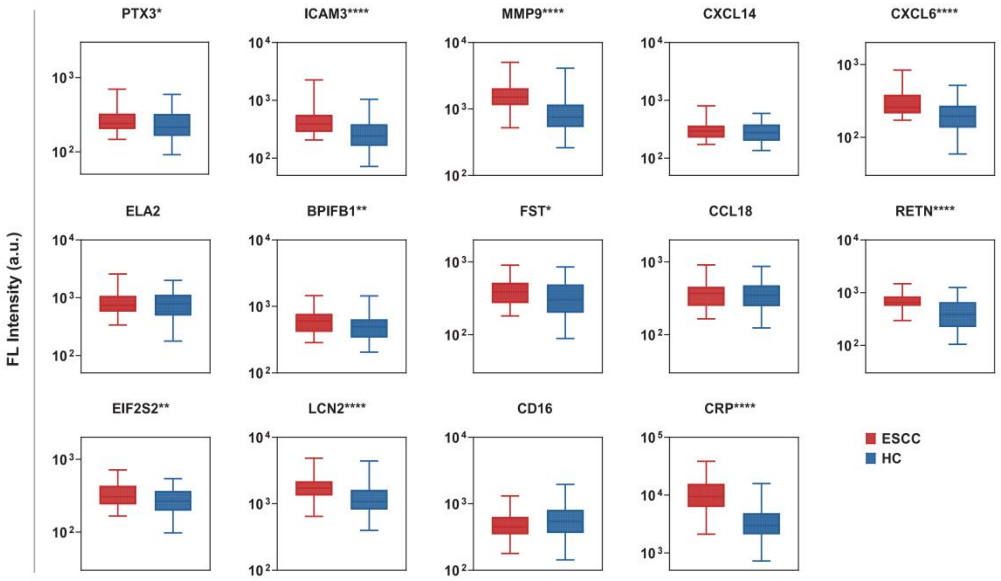

Figure S14. Box plot comparing the mean expression levels of 14 proteins in ESCC and HC in cohort 1.

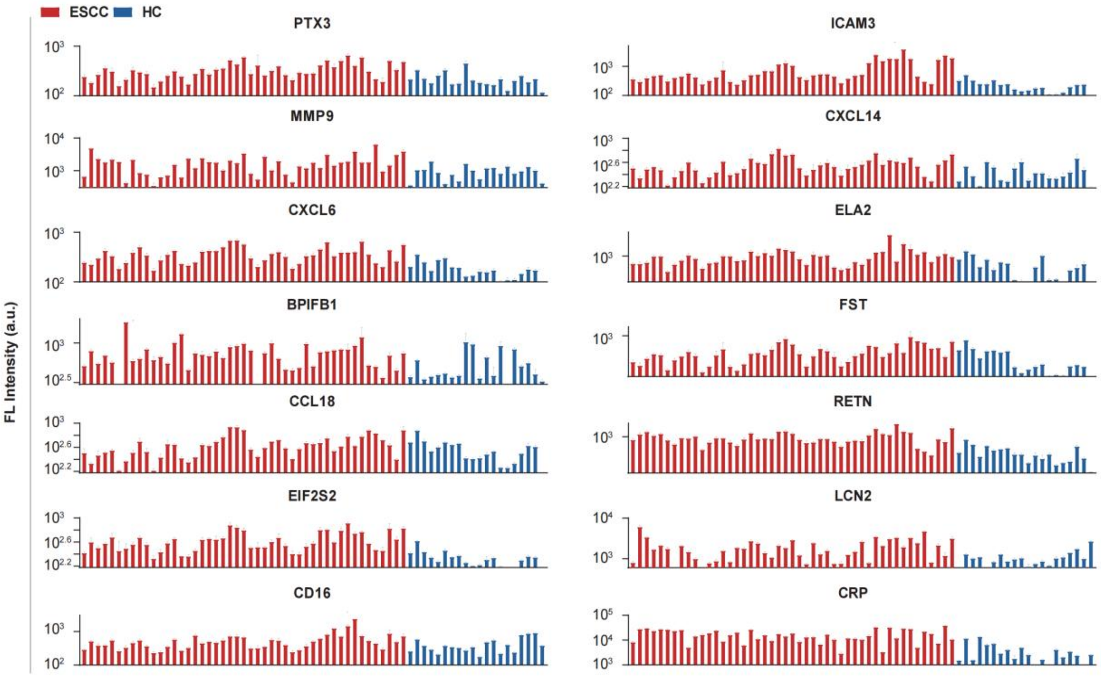

Figure S15. Bar chart of the fluorescence values for the detection of 14 proteins in cohort 2.

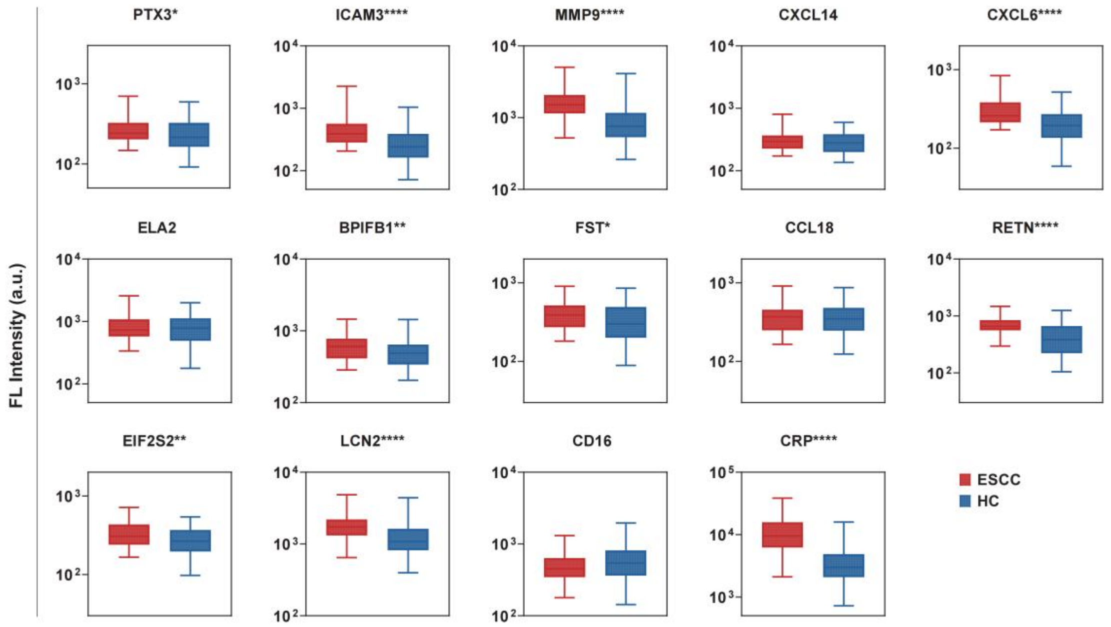

Figure S16. Box plot comparing the mean expression levels of 14 proteins in ESCC and HC in cohort 2.

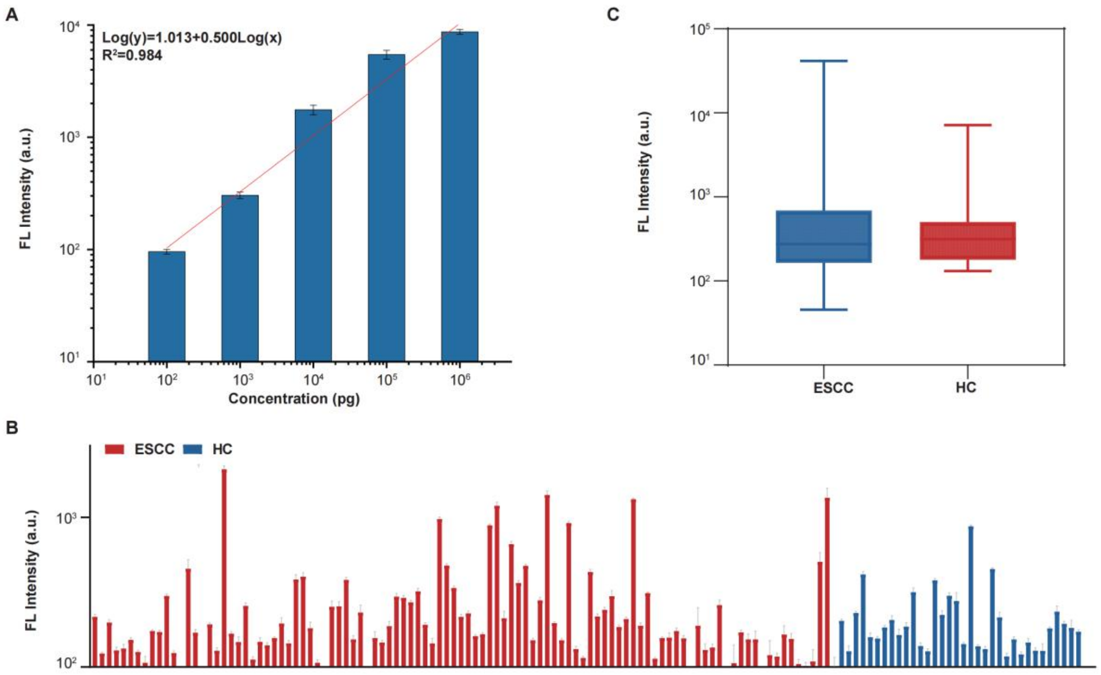

Figure S17. Detection of SCC. A. Standard curve for SCC detection on the chip. B. Bar chart of the fluorescence values for SCC detection. C. Box plot comparing the mean SCC levels detected in ESCC and HC.

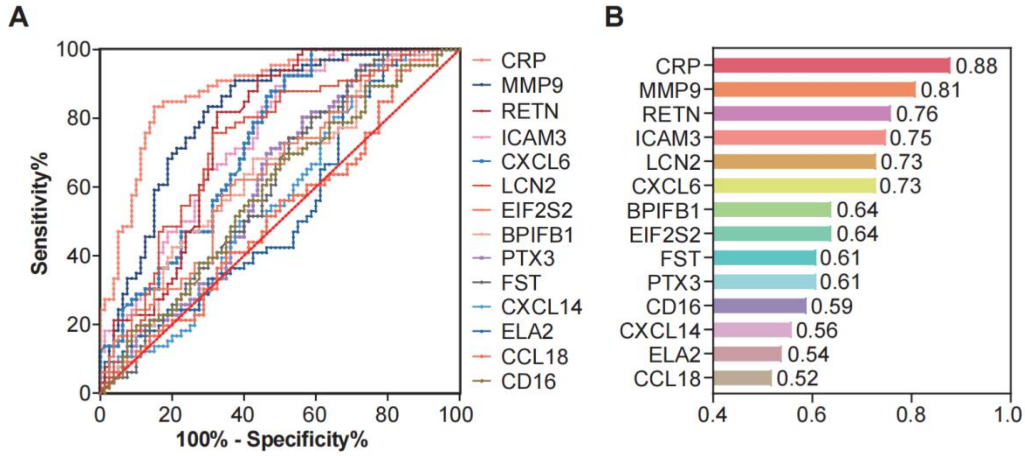

Figure S18. ROC assessment of 14 biomarkers. A. ROC curve of 14 individual biomarkers for diagnosing ESCC. B. Bar chart displaying the AUC values of individual biomarkers for ESCC.

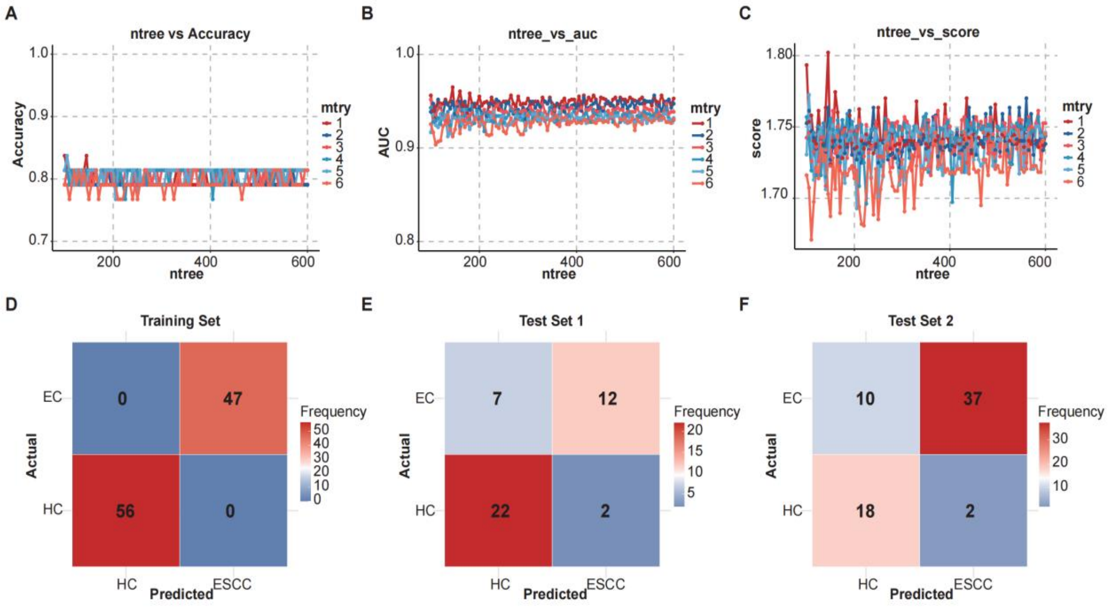

Figure S19. Parameter selection and results presentation for the construction of the 14-DM model. A. Relationship between the selection of ntree and mtry parameters and accuracy. B. Relationship between the selection of ntree and mtry parameters and AUC. C. Relationship between the selection of ntree and mtry parameters and the combined scores of accuracy and AUC. D. Confusion matrix of the 14-DM model in the training set. E. Confusion matrix of the 14-DM model in the training set. E. Confusion matrix of the 14-DM model in Test Set 1. F. Confusion matrix of the 14-DM model in Test Set 2.

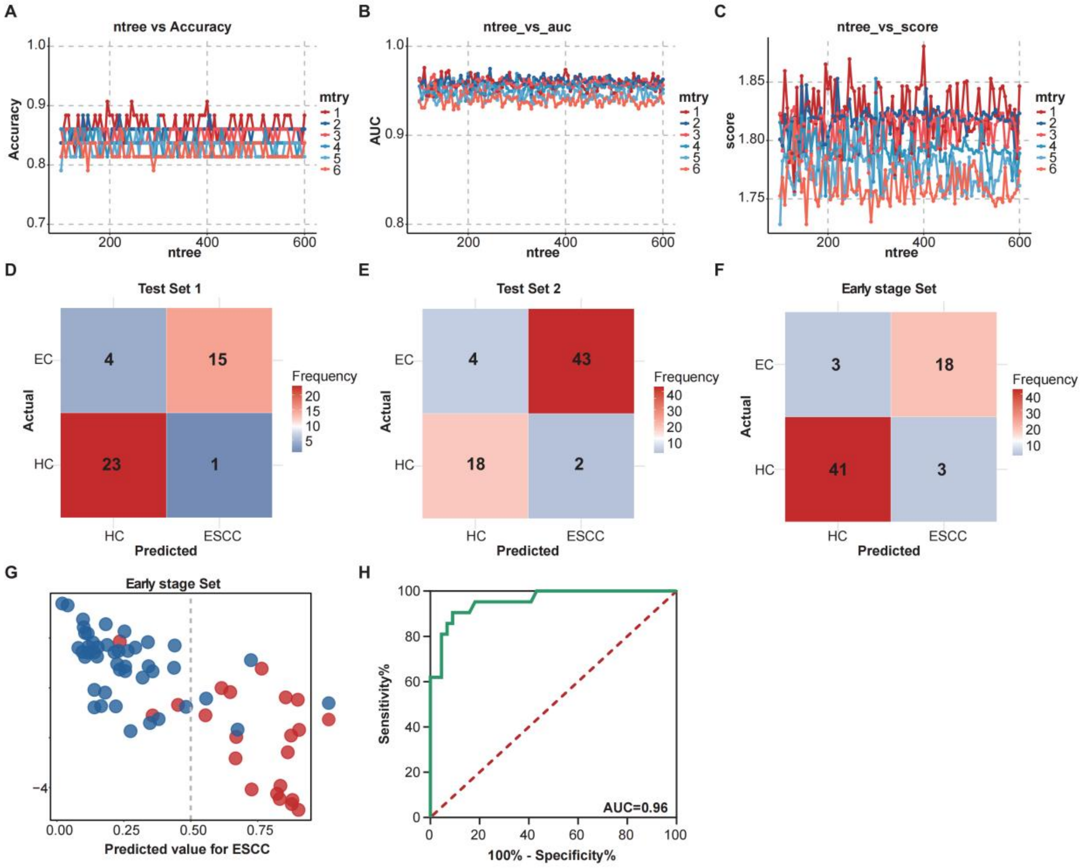

Figure S20. Parameter selection and results presentation for the construction of the 9-DM model. A. Relationship between the selection of ntree and mtry parameters and accuracy. B. Relationship between the selection of ntree and mtry parameters and AUC. C. Relationship between the selection of ntree and mtry parameters and the combined scores of accuracy and AUC. D. Confusion matrix of the 9-DM model in Test Set 1. E. Confusion matrix of the 9-DM model in Test Set 2. F. Confusion matrix of the 9-DM model in Early stage Set. G. The 9-DM model predicts I-II stage ESCC (red) versus HC (blue). The dashed line indicates a cutoff value of 0.50 for separately predicting HC (left) and I-II stage ESCC (right). H. ROC curves for the 9-DM model in diagnosing I-II stage ESCC.

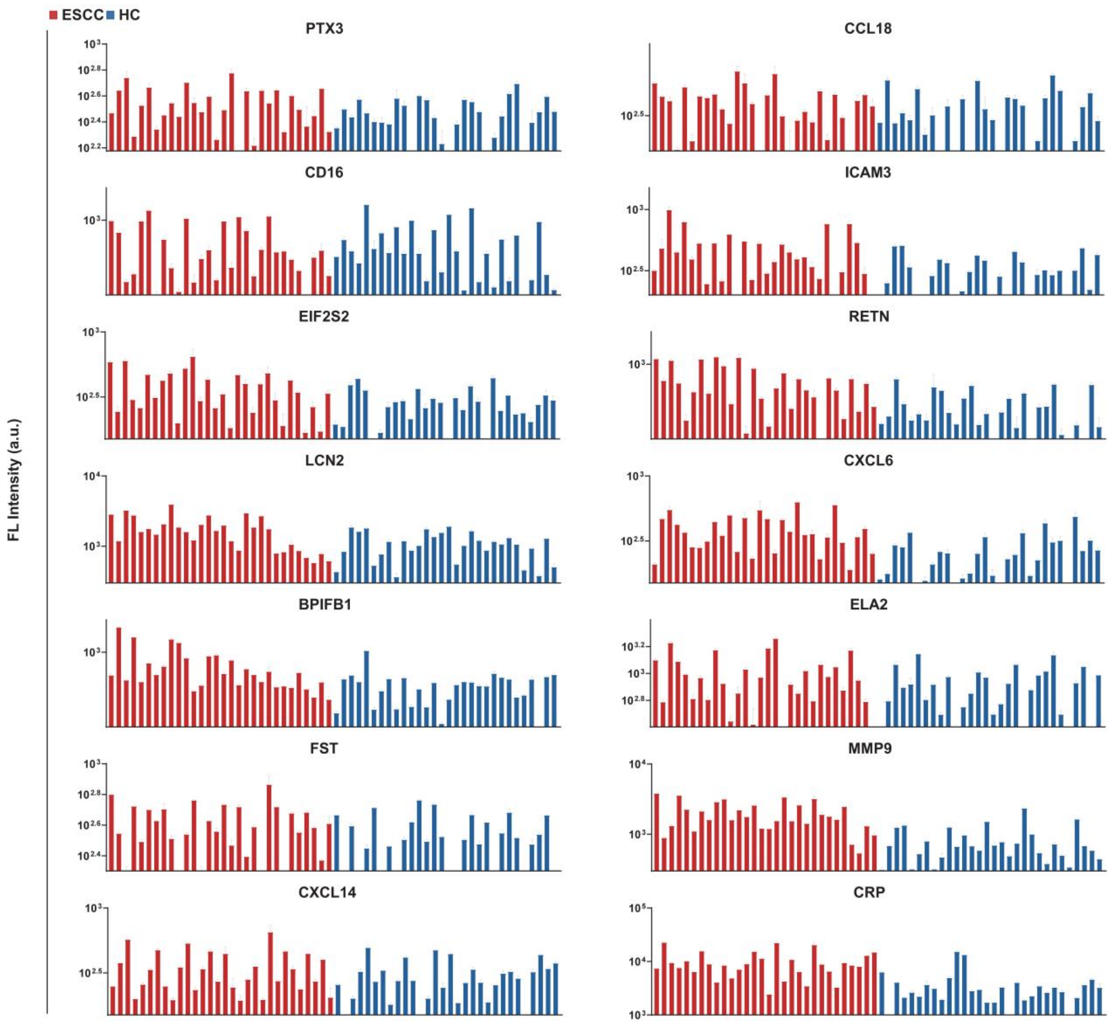

Figure S21. Bar chart of the fluorescence values for the detection of 14 proteins in cohort 3.

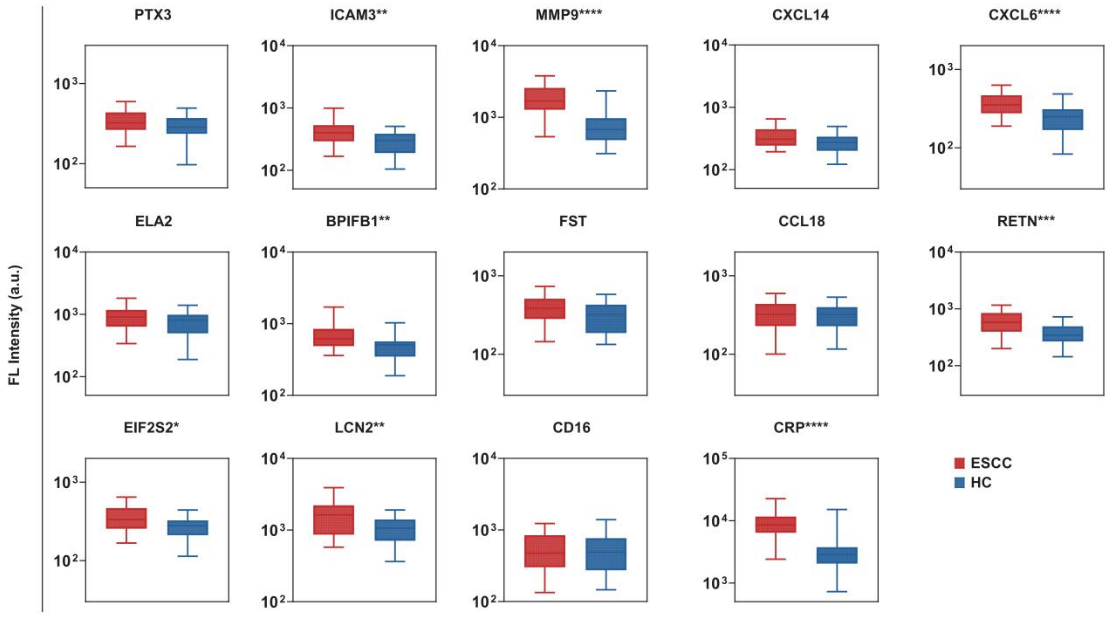

Figure S22. Box plot comparing the mean expression levels of 14 proteins in ESCC and HC in cohort 3.

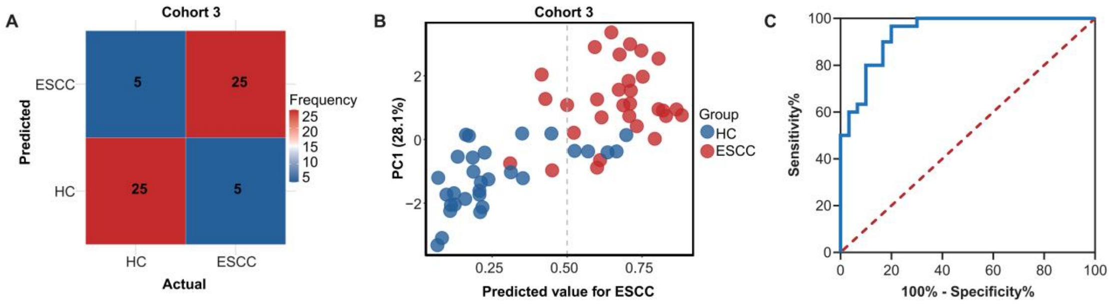

Figure S23. 9-DM model in cohort 3. A. Confusion matrix of the 9-DM model in the cohort 3. B. The 9-DM model predicts ESCC (red) versus HC (blue) in cohort 3. The dashed line indicates a cutoff value of 0.50 for separately predicting HC (left) and ESCC (right). C. ROC curves for the 9-DM model in diagnosing ESCC in cohort 3.

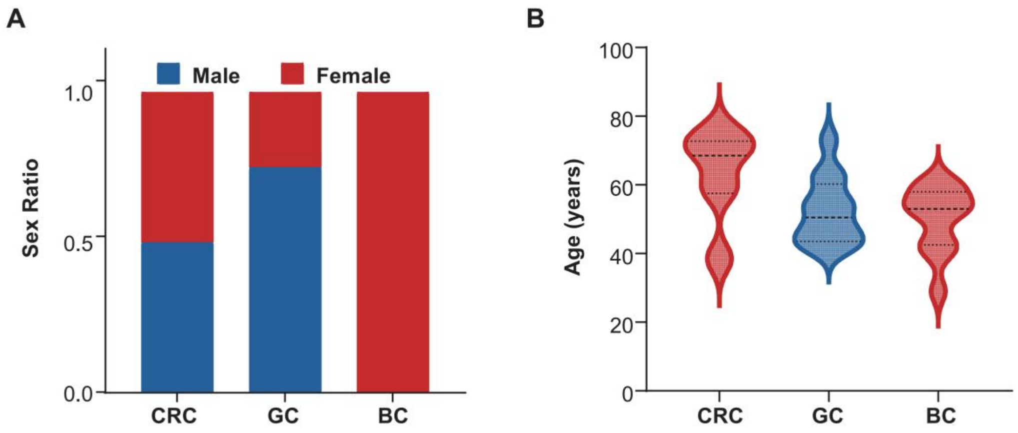

Figure S24. Basic information on samples from other cancer types. A. Bar chart displaying the gender ratio of patients with colorectal cancer (CRC), gastric cancer (GC), and breast cancer (BC). B. Violin plot showing the age distribution of patients with CRC, GC, and BC.

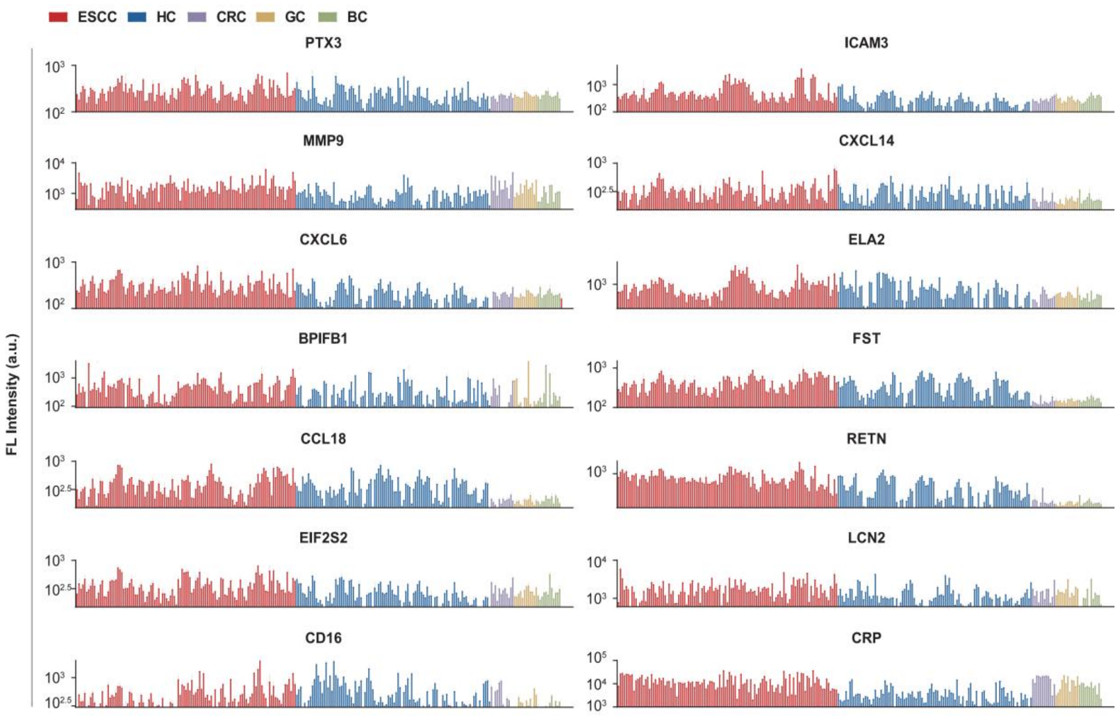

Figure S25. Bar chart displaying the fluorescence values of detections including other cancer types.

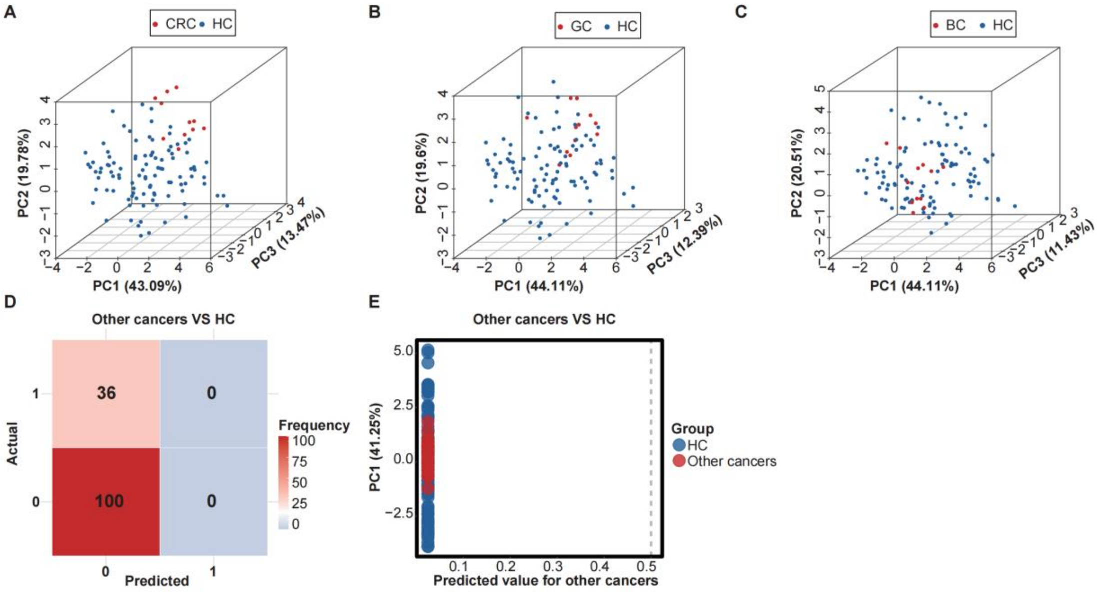

Figure S26. Presentation of machine learning results for other cancer types. A. PCA plot of 9 proteins in serum small EVs from CRC and HC. Each point represents a sample, with blue points indicating HC samples and red points indicating CRC samples. The % value represents the explained variance. B. PCA plot of 9 proteins in serum small EVs from GC and HC. Each point represents a sample, with blue points indicating HC samples and red points indicating GC samples. The % value represents the explained variance. C. PCA plot of 9 proteins in serum small EVs from BC and HC. Each point represents a sample, with blue points indicating HC samples and red points indicating BC samples. The % value represents the explained variance. D. Confusion matrix of other cancer types and HC in the 9-DM model. E. The 9-DM model predicts other cancer (red) versus HC (blue). The dashed line indicates a cutoff value of 0.50 for separately predicting HC (left) and other cancer (right).

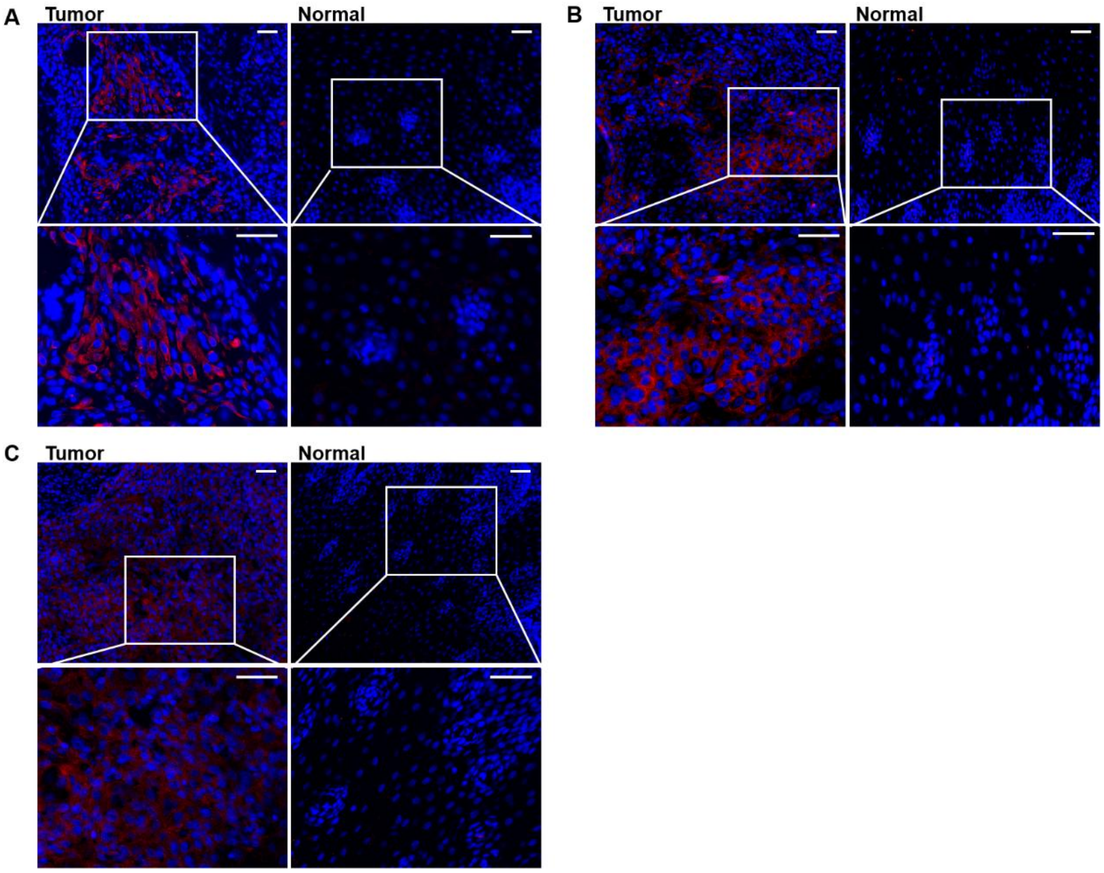

Figure S27. Comparative immunofluorescence analysis of candidate biomarkers. Representative images show A PTX3, B FST, and C EIF2S2 expression patterns in tumor tissues (left panels) versus paired adjacent normal tissues (right panels). Nuclei counterstained with DAPI (blue). Scale bars: 50 μm.

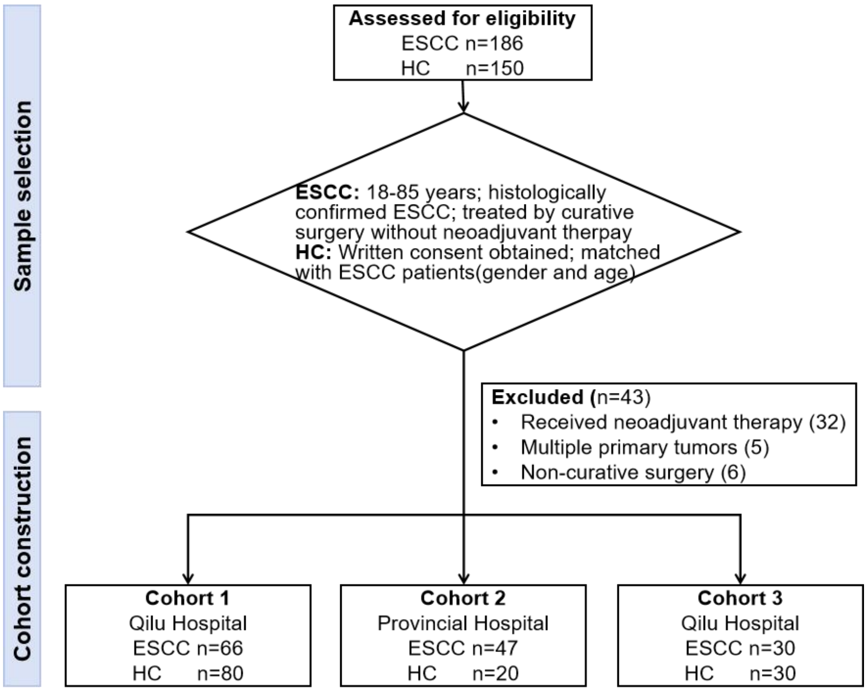

Figure S28. Flowchart of cohort screening and construction.

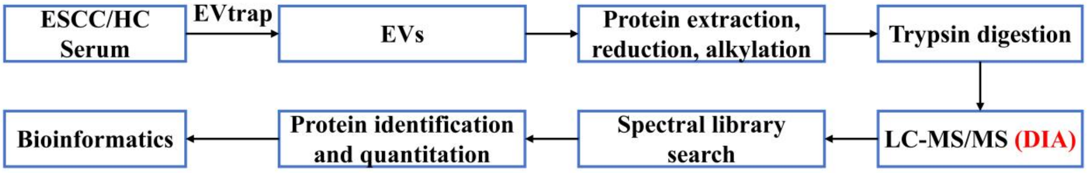

Figure S29. Schematic of the workflow for 4D-DIA mass spectrometry analysis of serum small EVs.

Serum small EVs were extracted using the EVtrap method. To 200 μL of serum sample, 1 mL loading buffer solution was added, followed by 25 μL of EVtrap beads. The mixture was inverted gently and incubated for 1 hour at room temperature (RT) with shaking. Following the incubation, the solution was separated using magnetic suction, and the supernatant was collected. A washing buffer solution was then added to the EP tube, and the beads were suspended by pipetting. The washing buffer solution containing the beads was transferred into the wells of a deep-well plate for subsequent operations. For small EVs solution samples, SDT buffer (4% SDS, 100 mM Tris-HCl, pH 7.6) was introduced directly to those samples. The lysates were subsequently sonicated and heated for 15 minutes. After centrifugation at 14,000 g for 40 minutes, the supernatant was measured using the BCA Protein Assay Kit (Bio-Rad, USA). A total of 20 μg protein from each sample was mixed with 5X loading buffer and heated for 5 minutes. The proteins were then resolved on a 4%-20% SDS-PAGE gel, running at a constant voltage of 180 V for 45 minutes. Protein bands were subsequently visualized by staining with Coomassie Blue R-250 . An equal volume from each sample in this experiment was combined into a single pooled sample for data-dependent acquisition (DDA) library generation and quality control. DTT was added to each sample at a final concentration of 10 mM, followed by mixing at 600 rpm for 1.5 hours at 37° C. Once the samples had cooled to RT, IAA was introduced to block reduced cysteine residues, bringing the concentration to 20 mM., The samples were kept in the dark and incubated for 30 minutes. Next, the samples were transferred to filters (Microcon units, 10 kDa). The filters were washed three times with 100 μl of UA buffer, followed by two washes with 100 μl of 25 mM NH4HCO3 buffer. Next, trypsin was added to the samples at a 1:50 (weight to weight) ratio, and the samples were incubated at 37 ° C for 15-18 hours (overnight), allowing the resulting peptides to be collected as a filtrate. The peptides were then desalted using C18 cartridges (Empore™ SPE Cartridges, C18 standard density, bed I.D. 7 mm, volume 3 ml, Sigma), concentrated by vacuum centrifugation, and reconstituted in 40 μl of 0.1% (v/v) formic acid. The peptide concentration was determined by measuring the UV light spectral density at 280 nm. For DIA experiments, indexed retention time (iRT) calibration peptides were added to the sample. The pooled digested peptides were then fractionated into 10 fractions using the Thermo Scientific™ Pierce™ High pH Reversed-Phase Peptide Fractionation Kit. Each fraction was subsequently desalted on C18 cartridges (Empore™ SPE Cartridges, C18 standard density, bed I.D. 7 mm, volume 3 ml, Sigma) and reconstituted in 40 μl of 0.1% (v/v) formic acid. The iRT-Kits (Biognosys) peptides were added to the samples before conducting the DDA analysis. The peptides from each sample were analyzed using a TIMSTOF mass

spectrometer (Bruker) connected to an Evosep One liquid chromatography system (Denmark) in DIA mode. The mass spectrometer acquired ion mobility MS spectra across a mass range of m/z 100-1700. Up to four windows were defined for each 100 ms TIMS scans based on the m/z-ion mobility plane. During PASEF MS/MS scanning, the collision energy was linearly ramped according to ion mobility, ranging from 20 eV at 1/K0 = 0.85 Vs/cm^2 to 59 eV at 1/K0 = 1.30 Vs/cm^2. DIA data were analyzed using Spectronaut™ 14.4.200727.47784, referencing the generated spectral library. Key software settings included dynamic iRT for retention time prediction, enabling interference correction at the MS2 level, and applying cross-run normalization. Results were filtered with a Q-value cutoff of 0.01, which corresponds to a false discovery rate of less than 1%.

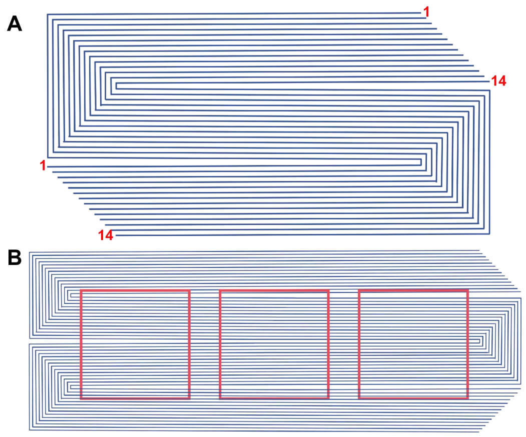

Figure S30. Micorchannels structure of the microprinting chip. A. Schematic of the zigzag layout of the 14 microchannels. B. Illustration of the detection unit and microchannel network. The red dashed box highlights the detection unit, each containing a complete 14-channel loop.

Table S1 Comparison of microfluidic biochips for the proteins detection

Name	Chip base	Limit of detection	Number of proteins	Number of samples	Sample volume	Readout
This work	Graphene oxide quantum dots	0.01-96 pg/mL	14	60	10 μL	Fluorescence
Perovskite QD based paper microfluidic device[1]	Nitrocellulose membrane,	95 pg/mL	2	1	20 μL	Fluorescence
3D microfluidic chip based on PCBs[2]	Photonic crystal beads	18.92 ng/ mL	3	1	50 μL	Fluorescence
Glass capillary-like microfluidic device[3]	Optimized ZnO nanorod arrays/ZIF-8 coating	0.01 pg/mL	1	1	unknown	Fluorescence
Quantum dot-based microfluidic protein chip[4]	QDs that are directly prepared in the aqueous phase (aqQDs)	2.5 pM	2	1	unknown	Fluorescence
Lys-AuNPs@MoS_2 nanocomposite self-assembled microfluidic[5] immunoassay biochip	Lys-AuNPs@MoS_2 Nanocomposite	0.24 pg/mL	6	60	2 μL	Fluorescence

Sequential microfluidic device[6]	/	47 pg/mL	1	1	4 μL	Electrochemical
Barcoded microchip[7]	/	10 pM	3	6	1 μL	Fluorescence
Microfluidic chip for cancer detection[8]	PMMA	20 ng/mL	1	1	unknown	Fluorescence
Snail-shaped microfluidic chip[9]	Silicon film	20 pg/mL	3	1	30 μL	fluorescence
A flux-adaptable pump-free microfluidics-based self-contained platform[10]	/	0.89 ng mL	4	1	20 μL	fluorescence
A frequency shift-based SERS microfluidic chip[11]	4-mercaptobenzoic acid -conjugated antibody functionalized gold nano-sheet substrate	0.38 pg/mL	2	3	/	SERS spectral

Table S2. Baseline characteristics of esophageal squamous cell carcinoma patients in cohort 1 , cohort 2 and cohort 3.

Characteristics	Esophageal squamous cell carcinoma			
	Cohort 1	Cohort 2	Cohort 3	p
**Overall**	66	47	30	
**Sex**				0.939
Male	53	38	25	
Female	13	9	5	
**Age**				0.513
<60	15	15	7	
≥60	51	32	23	
**Smoke**				0.427
Yes	37	29	21	
No	29	18	9	
**Drink**				0.187
Yes	33	26	21	
No	33	21	9	
**Lymph node metastasis**				0.137
Yes	36	33	15	
No	30	14	15	
**Stage**				0.112
I-II	32	15	16	
III-IV	34	32	14	
**Grade of Differentiation**				
G1	3	4	2	0.056
G2	25	15	13	
G3	26	14	15	
unknown	12	14	0	

References

[1] S. C.G, C. H. Ravikumar, R. G. Balakrishna, *Chemical Engineering Journal* **2023**, *464*, 142581.

[2] N. Chang, J. Zhai, B. Liu, J. Zhou, Z. Zeng, X. Zhao, *Lab Chip* **2018**, *18*, 3638.

[3] D. Zhao, Z. Wu, J. Yu, H. Wang, Y. Li, Y. Duan, *Chemical Engineering Journal* **2020**, *383*, 123230.

[4] M. Hu, J. Yan, Y. He, H. Lu, L. Weng, S. Song, C. Fan, L. Wang, *ACS Nano* **2010**, *4*, 488.

[5] J. Qiu, P. Jiang, C. Wang, Y. Chu, Y. Zhang, Y. Wang, M. Zhang, L. Han, *Analytical ChemistryAnalytical ChemistryAnal. Chem.* **2022**, *94*, 4720.

[6] S. Boonkaew, K. Szot-Karpińska, J. Niedziółka-Jönsson, B. Pałys, M. Jönsson-Niedziółka, *Sensors and Actuators B: Chemical* **2023**, *397*, 134659.

[7] Y. Zhang, J. Sun, Y. Zou, W. Chen, W. Zhang, J. J. Xi, X. Jiang, *Anal. Chem.* **2015**, *87*, 900.

[8] C. Sun, H. You, N. Gao, J. Chang, Q. Gao, Y. Xie, Y. Xie, R. X. Xu, *RSC Advances* **2020**, *10*, 39779.

[9] B. Yin, X. Wan, C. Qian, A. Sohan, S. Wang, T. Zhou, *Front Chem* **2021**, *9*, 741058.

[10] B. Dai, C. Yin, J. Wu, W. Li, L. Zheng, F. Lin, X. Han, Y. Fu, D. Zhang, S. Zhuang, *Lab Chip* **2021**, *21*, 143.

[11] Y. Huang, Z. Liu, X. Qin, J. Liu, Y. Yang, W. Wei, *Analyst* **2023**, *148*, 3295.
